# Synthesis, purification and characterization of *Plectonema* derived AgNPs with elucidation of the role of protein in nanoparticle stabilization†

**DOI:** 10.1039/d1ra08396a

**Published:** 2022-01-18

**Authors:** Almaz Zaki, Md. Nafe Aziz, Rakhshan Ahmad, Irshad Ahamad, M. Shadab Ali, Durdana Yasin, Bushra Afzal, Syed Mansoor Ali, Anita Chopra, Vijay Hadda, Pooja Srivastava, Raj Kumar, Tasneem Fatma

**Affiliations:** Department of Biosciences, Jamia Millia Islamia New Delhi India fatma_cbl@yahoo.com aziz.nafe@gmail.com; Department of Biotechnology, Jamia Millia Islamia New Delhi India; Institute of Nuclear Medicine & Allied Sciences (INMAS), Defense Research & Development Organization (DRDO), Government of India New Delhi India; Lab Oncology, All India Institute of Medical Sciences New Delhi India; Department of Pulmonary, Critical Care and Sleep Medicine, All India Institute of Medical Sciences New Delhi India

## Abstract

Driven by the need to biosynthesize alternate biomedical agents to prevent and treat infection, silver nanoparticles have surfaced as a promising avenue. Cyanobacteria-derived nanomaterial synthesis is of substantive interest as it offers an eco-friendly, cost-effective, sustainable, and biocompatible route for further development. In the present study optimal conditions for synthesis of silver nanoparticles (AgNPs) were 1 : 9 v/v [cell extract: AgNO_3_ (1 mM)], pH 7.4, and 30 °C reaction temperatures. Synthesis of nanoparticles was monitored by UV-vis spectrophotometry and the maximum absorbance was observed at a wavelength of 420 nm. SEM with EDX analysis confirmed 96.85% silver by weight which revealed the purity of AgNPs. TEM & XRD analysis exhibited a particle size of ∼12 nm with crystalline nature. FTIR analysis confirmed the presence of possible biomolecules involved in the synthesis and stabilization of AgNPs. Decapping of AgNPs followed by SDS-PAGE, LCMS and MALDI TOF analysis elucidates the proteinaceous nature of the capping and stabilizing agent. Cyanobacterial-derived capped AgNPs showed more cytotoxicicity towards a non-small cell lung cancer (A549) cell line, free radical scavenger and an antimicrobial than de-capped AgNPs. In addition they showed significant synergistic characteristics with antibiotics and fungicides. The test revealed that the capped AgNPs were biocompatible with good anti-inflammatory properties. The blend of antimicrobial and biocompatible properties, coupled with their intrinsic “green” and facile synthesis, made these biogenic nanoparticles particularly attractive for future applications in nanomedicine.

## Abstract 2

Illuminating the role of protein on the surface of cyanobacterial derived capped, decapped AgNPs and its biomedical application.

## Graphical abstract

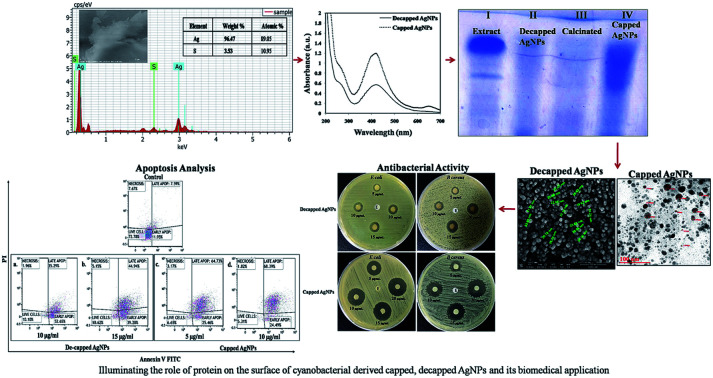

## 1. Introduction

Microbial infections caused by medical devices such as catheters and traumatic and surgical wound dressing pose a persistent threat and an overarching challenge to human health, despite the pioneering breakthroughs in antibiotics and antiseptics.^1^ Antibiotics use & misuse against microbial infection caused an outbreak of antibiotic resistance. 1.7 million cases and 100 000 deaths per annum were reported in the United States alone, and Gram-negative bacilli were the most common nosocomial pathogens.^2^ Increased antibiotic resistance of several pathogenic bacteria has compelled scientists to develop alternate anti-bacterial agents with higher potentials. The utilization of AgNPs can be particularly advantageous compared to their bulk counterpart due to their high surface area to volume ratio that provides better contact with microorganisms. AgNPs are non-toxic to human cells at low concentrations and are considered as safe antimicrobial agent.^3^ AgNPs can interact with the ligands and macromolecules of the microbial cell, causing a broad spectrum of bactericidal and fungicidal activities.^4^ Synthesis of AgNPs involves different chemical and physical methods, but the hazardous effects of their by-products and high cost are significant concerns.^5^ Naturally available resources like viruses, bacteria, cyanobacteria, fungi, algae, plants and biochemicals isolated from them like proteins, lipids, carbohydrates and secondary metabolites have been used in the green synthesis of nanoparticles. These provide intrinsic protein capping and stabilizing potential to the nanoparticles.^6–10^ Manipulation at various levels such as particle size, morphology, surface charge, coating and oxygen availability have been considered as important parameters to control and modulate the anti-bacterial activity of AgNPs. Among these parameters, surface coating (or functionalization) is the most crucial factor determining the nanoparticle–microbe interactions. Hence, studies targeted to understand the dynamic behavior of nanoparticles coatings are highly informative for designing efficient anti-bacterial formulations of AgNP.^11^

In the present study, an attempt was made to understand the nature and role of capping agents (*e.g.* protein) on the surface of nanoparticles of biogenic AgNPs [decapped AgNPs (SDS–protein complex), calcinated decapped AgNPs and capped AgNPs] derived from extracellular cyanobacterial *Plectonema* extracts and to define their role in bioactivity (interactions). For establishing the antimicrobial efficacy of synthesized biogenic AgNPs, the comparative anti-bacterial potential of both protein-capped and de-capped AgNPs was assessed. The adequate protein capped nanoparticles were further analyzed for their antioxidants, anti-bacterial, antifungal, synergistic, anti-inflammatory, biocompatibility and cytotoxic activity establishing their medical significance.

## 2. Materials and methods

### 2.1. Chemicals and microbial cultures

All the analytical grade chemicals purchased from Himedia, Sigma Aldrich and Merck Pvt. Ltd., India.

Cyanobacterial strain *Plectonema* sp. NCCU 204 was procured from Indian Agricultural Research Institute, New Delhi, India and was maintained in conical flasks (1000 mL) with BG-11.^12^ They were illuminated with 20 W Philips fluorescent tubes providing a light intensity of 2000 ± 200 lux for 12:12 hours light and dark cycles. Photobioreactor (FMT 150/3000-RW-PSI) (Photon Systems Instruments, Czech Republic) was used for large-scale biomass production. Sub-culturing was done at regular intervals. The bacterial strains *Escherichia coli* (MCC2412), *Bacillus cereus* (MCC 2243) and *Staphylococcus aureus* (MCC 2408) and the fungal strains *Candida albicans* (MCC-1151), *C. glabrata* (MCC-1432) were obtained from National Centre for Microbial Resource Pune, India and *Klebsiella pneumoniae* (KJ 938546) from Amity University, Noida, India.

### 2.2. Synthesis and characterization of capped AgNPs from *Plectonema* sp. NCCU-204 cell extract

After screening the 30 cyanobacterial strains for the synthesis of AgNPs *Plectonema* sp. was taken for further optimization and studies (details given in ESI Sections S1 & S2).† For cell extract preparation, 30 mL ddH_2_O was added to *Plectonema* sp. biomass (6 g) and homogenized. Then sonicated for 10 min and kept at 100 °C in the water bath for 10 min in 100 mL Erlenmeyer flask. After cooling to room temperature, the supernatant was filtered out with Whatman filter paper No. 1 and centrifuged at 6000 rpm for 10 min. Synthesis of AgNPs was carried out by the addition of 10 mL aqueous cell extract to 90 mL AgNO_3_ (1 mM) solution, followed by incubation at 30 ± 1 °C, pH 7.4 for 24 h under 2000 ± 100 lux.

Change in color of a reaction mixture (colorless to reddish-brown) was the first indication for the synthesis of AgNPs and these changes in optical properties were monitored quantitatively by scanning the spectra between 300–700 nm of wavelengths using UV-vis spectrophotometer. The purification of obtained nanoparticles was done through washing with double distilled water, organic solvents (acetone/ethanol) and centrifugation further characterization (XRD, EDX, SEM, TEM, DLS & zeta analysis and FTIR) was done. X-ray diffraction technique (XRD) having K-beta filter with X-ray 1.54056 Å with 30 mA of tube current and a voltage of 40 kV with scanning speed of 4° min^−1^ and the data was recorded in different 2*θ* angles ranging between 2 to 80° was adopted. The particle size (*D*) was determined using Scherrer equation (eqn (1))1
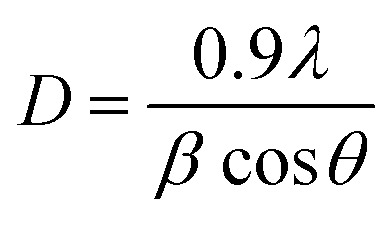
where ‘*λ*’ is wavelength of X-ray (0.1541 nm), ‘*β*’ is FWHM (Full Width at Half Maximum), ‘*θ*’ is the diffraction angle and ‘*D*’ is particle diameter size.

Energy dispersive X-ray (EDX Oxford Instrument, UK) was performed to check the presence of elemental silver inside the biologically synthesized nanoparticles, with an acquisition time ranging from 60 to 100 s and an accelerating voltage of 20 kV. The morphology and average size of the synthesized nanoparticles were further analyzed by SEM (Nova Nanosem-450 FEI, USA), HR-TEM (Philips, EM-410LS, JEOL, Japan). To perform the DLS and Zeta analysis (Nano Zetasizer system, Malvern Instruments) for particles size and stability, the sample loaded into quartz micro-cuvette, and measurement was taken. To identify the possible capping and stabilizing agents involved in the synthesis of AgNPs, FTIR (Varian 7000 FTIR USA) analysis was carried out in the range of 400–4000 cm^−1^ at a resolution of 4 cm^−1^ with KBr pellet as a reference.

### 2.3. Elucidation of protein in capped AgNPs

For the preparation of de-capped AgNPs through SDS treatment and calcinations, the protocol of Jain *et al.*, (2015) and Mathivanan *et al.*, (2019) respectively was adopted with slight modifications.^13,14^ For decapping by SDS treatment, the synthesized ethanol-washed protein-capped AgNPs solution was centrifuged at 12 000 rpm for 20 min. The pellet was suspended in 1% (w/v) sodium dodecyl sulphate (SDS) and boiled in a water bath for 30 min to detach the protein shell from nanoparticles, followed by centrifugation at 12 000 rpm for 20 min. The supernatant containing the unreacted SDS and SDS–protein complex was analyzed for the presence of proteins by measuring the UV-visible absorption spectrum. The resulting pellet was boiled in 1 mL of Tris–Cl (pH 8.0) in water bath for 10 min to eliminate the possibility of SDS binding to the nanoparticles. To ensure the complete removal of SDS, dialysis was performed using the dialysis cellulose membrane of pore size 10 kDa against Milli-Q water with four water changes. Ethanol washed AgNPs were calcinated at 100 °C for 30 min. The obtained de-capped AgNPs were characterized using Fourier transform infrared spectroscopy (FTIR) and UV-visible spectroscopy.

Total protein estimation (details given in ESI Section S9†) of aqueous cell extract (ACE) and capped AgNPs was done by modified method of Lowry *et al.*, (1951).^16^ Then SDS-PAGE analysis of ACE, capped AgNPs, de-capped AgNPs and calcinated AgNPs was performed to determine the protein profile as described by Laemmli, (1970).^15^ (details given in ESI Section S7).†

### 2.4. Biomedical application of capped AgNPs

#### 2.4.1. Antimicrobial and synergistic activity of capped AgNPs

To assess the anti-bacterial efficacy of biologically synthesized AgNPs, disc diffusion method or Kirby–Bauer method was performed against Gram-negative (*Escherichia coli*, *Klebsiella pneumonia*) as well as Gram-positive bacteria (*Bacillus cereus*, *Staphylococcus aureus*) on Mueller–Hinton agar plates.^17,18^ Antifungal activity was also determined against *Candida albicans* and *Candida glabrata* following standard guidelines of CLSI 2008 ^19^ (details given in ESI Section S4).†

Further antimicrobial activities of AgNPs were evaluated using the broth dilution technique according to the standard protocol of NCCLS (CLSI, 2008). Different concentration of AgNPs (200 μg mL^−1^ to 0.39 μg mL^−1^), streptomycin and fluconazole (200 μg mL^−1^ to 0.39 μg mL^−1^) as positive control, were placed into 96-well plate in a final volume of 100 μL. The test pathogens were harvested and their turbidity was assessed according to the McFarland 0.5 standard. Then, 100 μL of cell cultures (approximately 2.5 × 10^3^ cells per mL) were placed into the 96-well microtitre plate (Tarson) and incubated at 37 °C for 24 h. After incubation, the growth/turbidity was recorded at 600 nm using a spectrophotometer. The lowest concentration of AgNPs at which no visible growth occurred represented its MIC value.

The antimicrobial synergistic activities of AgNPs in combination with the standard antibiotic/fungicides were evaluated by the checkerboard assay.^20^ A microtitre plate was inoculated with 50 μL of AgNPs (200 μg mL^−1^ to 1.56 μg mL^−1^) and 50 μL of standard antibiotic/fungicides (100 μg mL^−1^ to 0.049 μg mL^−1^) concentration. Each well was inoculated with 100 μL of microbial suspension to make up the final volume 200 μL. The obtained checkerboard plates were incubated at 37 °C for overnight. The fractional inhibitory indexes (FIC) were calculated according to the eqn (2).2
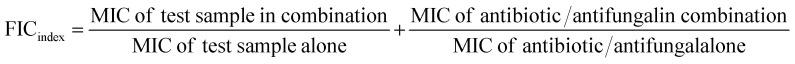
where, synergy and antagonism were defined by FICI ≤ and >4, respectively. Synergy was defined by FICI < 0.5, partially synergistic were defined by 0.5 < FICI < 1, whereas indifferent was defined by FICI ≤ 4.^17^

#### 2.4.2. *In vitro* antioxidant activities of capped AgNPs

The standard protocols were adopted for determining antioxidant activity [phosphomolybdenum assay, DPPH (1,1-diphenyl-2-picryl-hydrazil), ABTS (2,2′-azino-bis (3-ethylbenzothiazoline-6-sulfonic acid), ferric reducing antioxidant power (FRAP), nitric oxide radical scavenging activity].^21–25^ The experiments were performed in triplicates (details given in ESI Section S5†) percentage of inhibition was calculated using the following formula:3
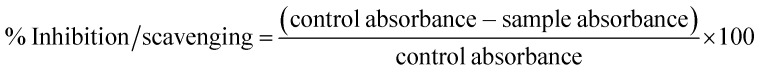


#### 2.4.3. Anti-inflammatory activity of capped AgNPs

To assess the anti-inflammatory activity modified method of Sakat *et al.* (2010) was performed.^26^ The reaction mixture consisted of 2 mL of AgNPs (25–175 μg mL^−1^) or ACE (25–300 μg mL^−1^) with 0.2 mL of 1% bovine serum albumin fraction was incubated at 37 °C for 20 min. Then heated at 57 °C for 20 min. After cooling, the turbidity of the reaction mixture was measured spectrophotometrically at 660 nm. Aspirin was used as the reference standard. The experiment was performed in triplicates. The percentage inhibition of protein denaturation was calculated using the eqn (3).

### 2.5. Biocompatibility assay of AgNPs

Biocompatibility of capped and de-capped AgNPs derived from *Plectonema* sp. was carried out, quantitatively by MTT assay and qualitatively by DAPI staining^27^(details given in ESI Section S10).†

#### 2.5.1. Annexin V FITC assay for apoptosis analysis

After treatment with AgNPs, we performed the apoptosis assay on A549 cell lines using APC-Annexin V/PI detection kit (BioLegend, USA: Cat no. 640932). Firstly, the cells were treated with different concentrations of AgNPs for 24 hours. Then, the cells were stained with APC conjugated Annexin V and PI as per the manufacturer's recommendation and then the samples were run on flow cytometry (Gallios, Beckman Coulter, USA) and analyzed by Kaluza analysis software (Beckman Coulter, USA).

### 2.6. Statistical analysis

All the experiments were carried out in triplicates (*n* = 3) and the values are expressed as means ± SD. Statistical analysis was done using OriginPro 8.5 (2011). Two-way ANOVA was performed to determine whether there are any statistically significant differences between the means of two or more independent groups. *P*-values <0.05 were regarded as significant.

## 3. Results and discussion

### 3.1. Synthesis, optimization, purification and characterization of AgNPs derived from *Plectonema* sp. NCCU 204 cell extract

**Fig. 1:**
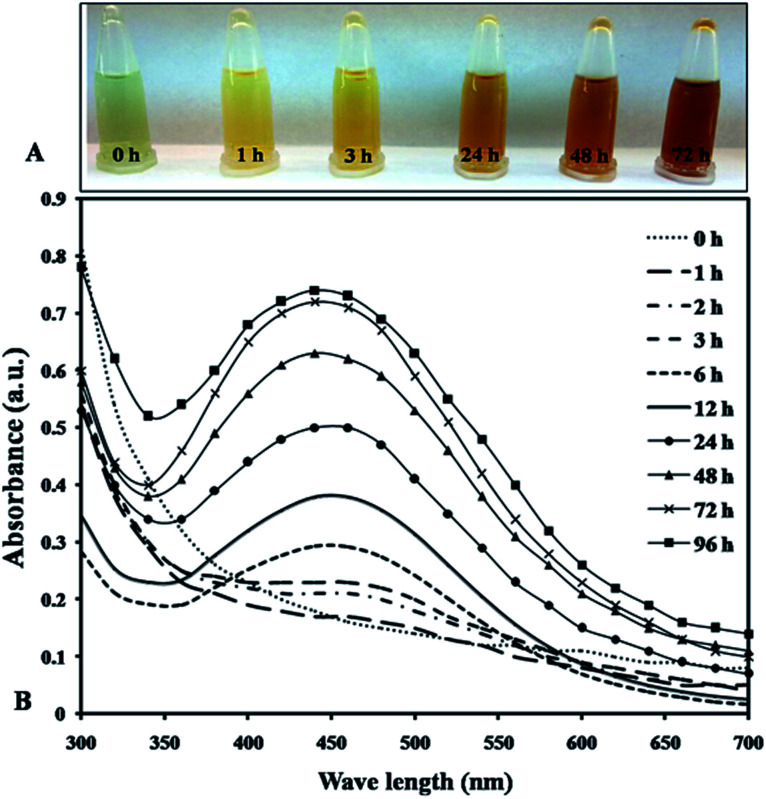
(A) Change in the intensity of the synthesized nanoparticles; (B) UV-vis spectra of synthesized AgNPs recorded at the different time interval.

Cell extract using optimized conditions (aqueous extracts preparation at 100 °C for 10 min, 1 mM AgNO_3_, pH 7.4 at 30 °C) taken to synthesized AgNPs resulted in color transition from greenish to yellowish brown indicating formation of nanoparticles over a 72 h period (Fig. 1A).^28^ According to Ali *et al.* 2011, used extract of *Oscillatoria* Willei NTDM01 to synthesize silver nanoparticles and suggested the involvement of proteins as a capping molecule for its stabilization.^29^ The surface plasmon resonance (SPR) was found to increase at 440 nm at different time interval indicating the synthesis of AgNPs. It was noted that the reduction of AgNO_3_ solution into AgNPs started within 1 h after the addition of AgNO_3_ solution into cell extract and completed at 72 h after that reaction saturation was observed (Fig. 1B). Ahamad *et al.*, 2021 while working with *Anabaena variabilis*, reported the minimum reduction time 1 h with absorption peak at 440 nm for an average size range of 11–15 nm during TEM analysis.^10^ So we observed that *Plectonema* sp. NCCU 204 stood out with least reduction time (30 min), smallest average size range (9–17 nm) with spherical in shape through SEM and thus used further studies (Fig. S1 & Table S1†).

**Fig. 2:**
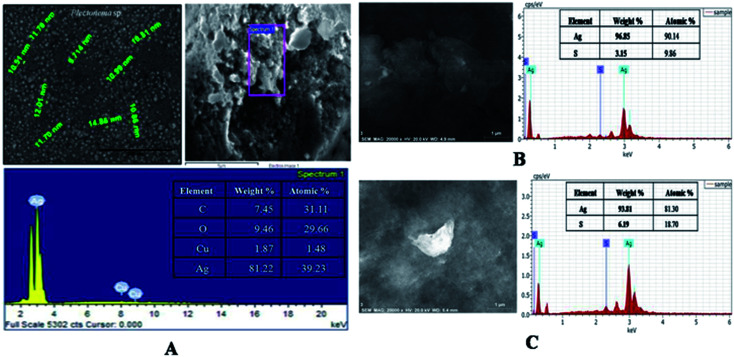
SEM with EDX of capped AgNPs (A) water washed, (B) ethanol washed and (C) acetone washed.

The optimized lyophilized biogenic synthesized AgNPs (Fig. S2†) were purified through washing with ddH_2_O, organic solvents (acetone and ethanol), dilution, centrifugation and filtration. The ddH_2_O washed AgNPs characterization through SEM with EDX analysis showed 81.22% Ag (wt%) and 39.23% Ag (at%) [Fig. 2A]. Purity of AgNPs further increased after washing with ethanol from 81.22% to 96.96% as Ag (wt%) and from 39.23% to 90.46% as Ag (at%) [Fig. 2B]. With acetone washing also, purity increased from 81.22% to 93.81% as Ag (wt%) and from 39.23% to 81.30% as Ag (at%) (Fig. 2C). Due to greater purity, the lyophilized ethanol washed AgNPs (capped AgNPs) were used for further studies. Similar observation was also reported by Licona *et al.*, (2019) where AgNPs synthesized from *Paulownia tomentosa* leaves were purified by ethanol.^30^

#### 3.1.1. Physico-chemical characterization of capped AgNPs

**Fig. 3:**
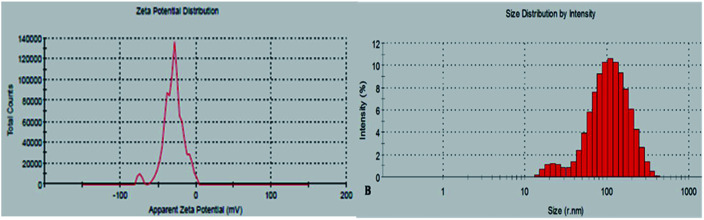
(A) Zeta potential; (B) DLS analysis of protein capped AgNPs, AgNPs.

The physico-chemical and biological properties of *Plectonema* cell extract derived AgNPs was done for finding probabilities of their future application. Zeta potential measurement was done to check the stability of synthesized protein capped AgNPs spectroscopically. Metal nanoparticles with large positive (>+30 mV) or negative (≥30 mV) charges tends to repel each other and do not show deposition, and provide stability to the nanoparticles. In case of low zeta potential values the particles aggregate and flocculates due to absence of repulsive force. The zeta potential of the protein capped AgNPs was found to be −29.7 mV (Fig. 3A). Raj *et al.* (2020) synthesized AgNPs from *Terminalia arjuna* leaf extract with zeta potential of −21.7.^31^ The more negative value of zeta potential of the *Plectonema* AgNPs suggested more stability of the nanoparticles probably due to presence of protein moieties.

Dynamic Light Scattering (DLS) was used to determine hydrodynamic sizes, polydispersities and aggregation effects of colloidal samples. The substances adsorbed on the surface of the nanoparticles (*e.g.*, stabilizers) and the thickness of the electrical double layer (solvation shell), moving along with the particle makes the size bigger in comparison with SEM and TEM microscopic techniques.^32^ The mean average size of the nanoparticles was found to be around 20 nm and 110 nm (Fig. 3B). The polydispersity index (PDI) of AgNPs was 0.212 which pointed out that these particles are moderately dispersed.^33^

**Fig. 4:**
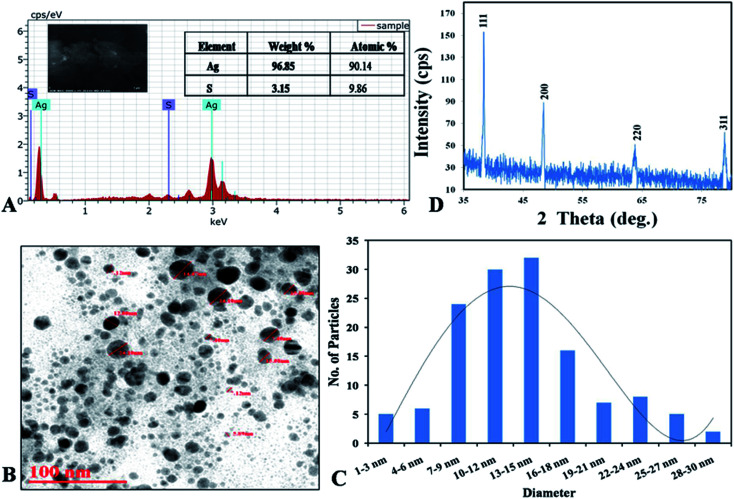
(A) XRD spectrum of capped AgNPs; (B) SEM with EDX graph with elemental percentage table; (C and D) TEM micrograph with histogram.

XRD analysis are basically used to determine the physio-chemical properties of the unknown materials.^34^ During analysis, the biosynthesized AgNPs showed crystalline nature (Fig. 4A). When the crystalline size decreases from bulk to nanoscale dimensions, the XRD peaks broaden.^35^ From our study the XRD showed that protein capped AgNPs formed are crystalline in nature with average size 20 nm close to particle size measured by TEM. Four peaks at 2*θ* values of 38.3, 48.44, 63.82 and 78.86 deg. corresponds to (111), (200), (220) and (311) planes of silver is observed and compared with the standard powder diffraction card of Joint Committee on Powder Diffraction Standards (JCPDS), silver file no. 04-0783. The XRD study confirmed that the resultants particles were in face centered cubic arrangement of atoms inside the AgNPs.

Eqn (3) SEM with energy dispersive X-ray (EDX) analysis was performed to confirm the presence of elemental silver inside the biologically synthesized nanoparticles. During the EDX spectral analysis of optical absorption band at 3 keV confirmed the presence of elemental silver in nanoparticles. Additional peaks represent for sulphur which occur due to presence in proteins and other biomolecules of capping agent of the AgNPs, comes inside the ddH_2_O while preparation of the samples. Similar results were also reported by Aziz *et al.* (2016).^7^ The weight percentage of silver element was found to be 96.85% as compared to another element present in the sample (Fig. 4B). Transmission electron microscopy (TEM) was carried out to observe the morphology and size of the biosynthesized AgNPs. The size of the nanoparticles was found to be in the range of 2–30 nm with an average size of 12 nm with spherical in shape (Fig. 4C and D).

### 3.2. Elucidation of protein in capped AgNPs derived from *Plectonema* sp. NCCU-204 cell extract

**Fig. 5:**
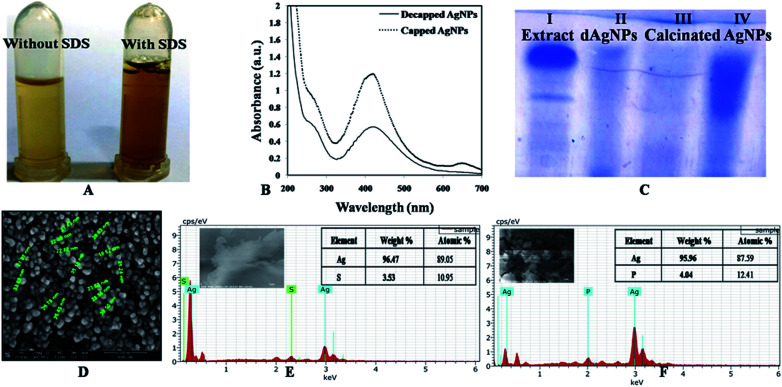
(A) Supernatant without and with SDS during decapping of ethanol washed AgNPs, (B) UV-visible absorbance decapped and capped AgNPs (Pellet dissolve in ethanol); (C) SDS PAGE of cell extract (I), decapped AgNPs (II) [SDS treated-dAgNPs], calcinated AgNPs (III) and capped AgNPs (IV); (D) SEM micrograph; (E) SEM with EDX of (ethanol washed) decapped AgNPs; (F) SEM with EDX of calcinated (ethanol washed) AgNPs.

**Fig. 6:**
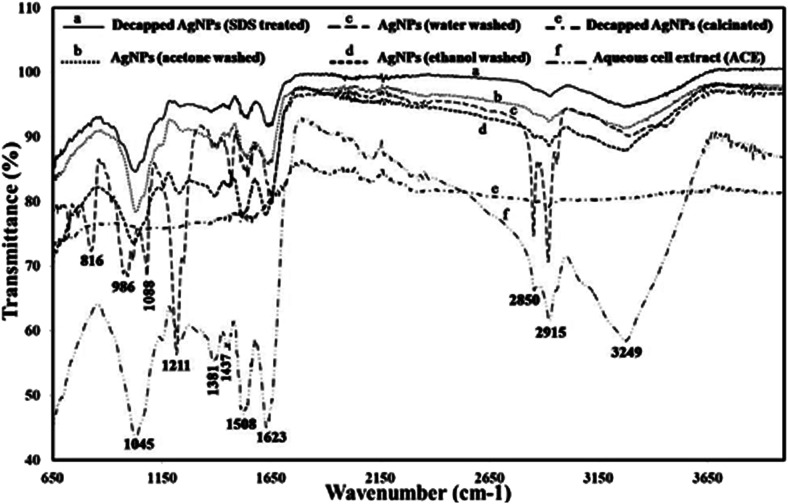
FTIR analysis of capped [(c) water, (b) acetone and (d) ethanol washed), decapped (ethanol washed- (a) SDS treated and (e) calcinated] AgNPs and (f) aqueous cell extract.

In order to see the effect of capping agent in bioactivity, decapping of the synthesized AgNPs was done by SDS and calcination. SDS was used for protein decapping from AgNPs as it result in detachment of surface bound protein. The absorbance of the above supernatant samples at 280 nm gave absorbance value at 0.4899 and 0.7794 respectively and, indicated that the protein was removed from the surface of AgNPs during decapping of nanoparticles. Any absorbance peak at 280 nm was observed in negligible amount in de-capped AgNPs pellet, suggesting that protein moieties were removed from the AgNPs after SDS treatment (Fig. 5A and B). Calcination was also done to remove the organic moieties present on the surface of AgNPs. SEM with EDX of the capped, decapped (SDS treated ethanol washed) and calcinated AgNPs showed 96.96% & 90.46%, 96.47% respectively as Ag (wt%) and 89.05% and 95.96% & 87.59 respectively as Ag (at%) (Fig. 5E and F). The size of the de-capped AgNPs was found to be in the range of 15–35 nm through SEM (Fig. 5D). Increase in the size of nanoparticles might be due to removal of capping or stabilizing agents present on the surface of nanoparticles that may have caused aggregation. During biogenic nanoparticles biosynthesis, enzymes and proteins play important role in synthesis and stability of the nanoparticles. Therefore, FTIR analysis aqueous cell extract (ACE), capped AgNPs (water, acetone & ethanol washed) as well as de-capped (SDS treated & calcinated) AgNPs was performed (Fig. 6 & Table S2†). The prominent spectral peaks detected were found at 3249, 2915, 2850, 1623, 1508, 1437, 1381, 1211 and 1045 cm^−1^. Spectral peak at 3249 cm^−1^ corresponds to (O–H) stretching vibrational frequency in phenols and alcohols. Vibrational peaks at 2850 and 2917 cm^−1^ were the characteristics of N–H stretching vibrational frequency for the amines functional group of proteins which was also reported by Isaac *et al.* (2013).^37^ Peaks at 1623 cm^−1^ corresponds to N–H bending of amines associated with amide linkage in peptide and proteins. Similar observation was also noticed by Castro *et al.*, (2013) in AgNPs synthesized from aqueous cell free extract of *Chondrus crispus* and *Spirogyira insignis*.^38^ Peak at 1508 cm^−1^ corresponds to N–O stretching vibrational frequency of nitro compound and 1437 cm^−1^ corresponds to O–H bending vibrational frequency of carboxylic acid. Peaks at 1381 cm^−1^, correspond to C–H bending of alkanes whereas 1211 and 1045 cm^−1^ C–N stretching vibration of the amine group. Gole *et al.* (2001) emphasized that negatively charged carboxylate group, amine group or cysteine residues present in the proteins may interact with metallic nanoparticles.^39^

In capped AgNPs three more peaks were detected at 816, 986, 1088 cm^−1^ which corresponds to C–H bending, C=C bending of mono-substituted alkene and C–O stretching of secondary alcohol. Two peaks (1045 & 1623 cm^−1^) of amines group with less intensity were also noticed that may be due to some impurities which were not detected during SDS PAGE analysis in de-capped AgNPs. FTIR of calcinated AgNPs any spectral peak could not be detected that may be because of decapping. Reduction in the intensities of the peaks (1045, 1508, 1623, 3249 cm^−1^) after biosynthesis of the AgNPs (as compared to aqueous cell extract) suggests that these biomolecules (mostly amino acids) along with the other reducing agents such as phenols and carbohydrates are responsible for reducing, capping and stabilization of AgNPs. Results of the present study agrees with the previous reports.^40,41^

Total protein content (TPC) was obtained from the calibration curve *y* = 0.0018*x* + 0.0692 (*R*^2^ = 0.9309) and was found to be 212.07 mg mL^−1^ for cyanobacterial aqueous extract and 163.39 mg mL^−1^ for protein capped AgNPs. Significantly lower TPC content of AgNPs indicated utilization of cellular extract proteins in AgNPs synthesis that possibly formed a covering layer of the nanoparticles. Due to protein capping, synthesized nanoparticles remain separated without agglomeration.^42^ El-Naggar *et al.* (2018) used phycobiliprotein to synthesize AgNPs.^43^ Ballottin *et al.* (2016) stablish the similar results and identified 8 proteins involved in the capping and stabilization of silver nanoparticles synthesized from *Aspergillus tubingensis*-isolated as an endophytic fungus from *Rizophora mangle*.^44^ SDS-PAGE analysis was performed for verifying the proteinaceous nature of the capping agent. Lane-I exhibited two protein bands in the aqueous cell extract (ACE) isolated from *Plectonema* sp. (Fig. 6C). Lane-II & III of de-capped SDS treated and calcinated AgNPs pellet exhibited no band proving complete removal of protein from the surface of the nanoparticles. Therefore, it can be said that the proteins act as a capping material and confer stability to AgNPs. While lane-IV with capped AgNPs treated supernatant exhibited proteins smear.

During LCMS-MALDI TOF analysis of the two protein bands of SDS-PAGE and their matching with the proteome database was done, upper protein band (of about 20 kDa), matched with Geranylgeranyl diphosphate reductase (Mascot top score of 88) of peptide mass fingerprint (PMF) of *Synechocystis* sp. (strain PCC 6803/Kazusa). Geranylgeranyl reductase catalyses the reduction of geranylgeranyl pyrophosphate to phytyl pyrophosphate is required for synthesis of chlorophylls, phylloquinone and tocopherols in cyanobacteria. Lower protein band (of about 12 kDa), matched with 2-isopropylmalate synthase (Mascot top score of 130) of peptide mass fingerprint (PMF) of *Prochlorococcus marinus* sub sp. pastoris (strain CCMP1986/NIES-2087/MED4). In cyanobacteria 2-isopropylmalate synthase catalyzes the condensation of the acetyl group of acetyl–CoA with 3-methyl-2-oxobutanoate (2-oxoisovalerate) to form 3-carboxy-3-hydroxy-4-methylpentanoate (2-isopropylmalate). This protein is involved in the pathway that synthesizes l-leucine from 3-methyl-2-oxobutanoate in cyanobacteria (details given in ESI Section S7).†

### 3.3. Biomedical application of capped AgNPs

#### 3.3.1. Antimicrobial activity of capped AgNPs

The anti-bacterial activity of ddH_2_O washed AgNPs was compared with purified capped AgNPs (ethanol washed) against *E. coli* (Gram negative) and *Bacillus cereus* (Gram positive). A significant increase in zone of inhibition was observed against both bacterium for ethanol washed AgNPs (Table 1 & Fig. S3†). Similar observation was also reported by Licona *et al.*, (2019)^30^ where AgNPs synthesized from *Paulownia tomentosa* leaves ethanol extract gave better anti-bacterial activity against Gram positive *Staphylococcus aureus* bacteria.

**Table 1:** Antibacterial activity of water washed and ethanol washed AgNPs

Amount of nanoparticles	Capped AgNPs		Capped AgNPs	
	Water washed		Ethanol washed	
	*E. coli*	*Bacillus cereus*	*E. coli*	*Bacillus cereus*
5 μg	9 ± 0.5 mm	11 ± 0.4 mm	19 ± 0.9 mm	12 ± 0.8 mm
10 μg	12 ± 0.6 mm	17 ± 0.7 mm	23 ± 1.5 mm	20 ± 1.5 mm
15 μg	14 ± 0.9 mm	19 ± 1.1 mm	25 ± 1.2 mm	23 ± 1.6 mm
20 μg	16 ± 1.2 mm	21 ± 1.5 mm	27 ± 1.4 mm	25 ± 1.8 mm

Further purified de-capped AgNPs (SDS treated ethanol washed) was compared with capped AgNPs. Capped AgNPs were more effective against *E. coli* and *Bacillus cereus* (Gram positive) than de-capped AgNPs (Fig. S6† & Table 2), the efficacy of de-capped AgNPs was effective against *E. coli* (Gram negative) observed during the present study as compared to *Bacillus cereus* is in complete agrement with the previous studies.^26,40^ A relatively thick and continuous peptidoglycan cell wall in Gram positive bacteria restrict the entry of de-capped AgNPs.^26^ However, the interactions of teichoic acid (which span the peptidoglycan layer) and side chains of amino acids of capped AgNPs may facilitate their possible entry in Gram positive bacterial species.^10^

**Table 2:** Antibacterial activity of capped and decapped (ethanol washed-SDS treated) AgNPs

Amount of nanoparticles	Decapped AgNPs		Capped AgNPs	
	*E. coli*	*Bacillus cereus*	*E. coli*	*Bacillus cereus*
5 μg	10 ± 0.6 mm	9 ± 0.5 mm	19 ± 0.9 mm	12 ± 0.8 mm
10 μg	13 ± 0.4 mm	11 ± 0.6 mm	23 ± 1.5 mm	20 ± 1.5 mm
15 μg	15 ± 0.7 mm	14 ± 0.9 mm	25 ± 1.2 mm	23 ± 1.6 mm
20 μg	16 ± 0.8 mm	17 ± 1.1 mm	27 ± 1.4 mm	25 ± 1.8 mm

#### 3.3.2. Synergistic activity

Synergistic action is used to describe an interaction of two antimicrobial agents or occasionally more than two, in which the effect produced by the drugs in combination is greater than their individual effects.^36^ The interaction index for each combination was determined by checkerboard methods. The fractional inhibitory concentration indexes (FICI) against pathogenic bacteria *B. cereus, S. aureus, E. coli, K. pneumoniae* with AgNPs and streptomycin. The FICI values obtained were 0.374 ± 0.12, 0.374 ± 0.08, 0.311 ± 0.04 and 0.374 ± 0.11 respectively (Table 3). Similar results were observed against *C. albicans* and *C. glabrata* with AgNPs and fluconazole correspondingly their FICI were 0.336 ± 0.06 and 0.312 ± 0.06 (Table 4). When the FIC index of the combination is equal to or less than 0.5, then the combinations are termed as synergistic; when FIC index falls between 0.5 and 4 it indicates no interaction between the two drugs, value above 4 indicates antagonism.^45^ In the present study, FICI value was less than 0.5 collectively, our results highlights the presence of synergistic interactions between AgNPs and antibiotic/fungicides combinations and opened the door for their use against multidrug resistant strains. The possible mechanism for the enhancement of antimicrobial activity using combination of AgNPs and antibiotics or antifungal agents is that the active functional groups of antibiotics such as hydroxyl and amino groups can be chelated by silver and thereby cover a considerable portion of the surface of AgNPs. According to Raj *et al.*, (2012) the AgNPs destroy the stability of lipopolysaccharides causing permeability of outer membrane and the peptidoglycan structure, which was immediate recognized and captured by antibiotics (*e.g.*, cephalexin), and the conjugation of antibiotics with silver nanoparticles makes the resistant strain to become sensitive to antibiotics.^46^ The individual effect of antimicrobial activity was also done using disc diffusion method on Mueller–Hinton agar plates (Fig. S4) [details given in ESI Section S4].† Thus during this study when AgNPs was combined with antibiotic/antifungal standards, significant synergistic effect was observed.

**Table 3:** Minimum inhibitory concentration (MIC) against pathogenic bacteria alone and in combination with antibiotic (streptomycin) along with interaction index. Experimentswere performed in triplicates; mean ± SD are shown

MIC (μg)			Interaction index			
Bacteria	AgNPs	Streptomycin (strep.)	FIC (μg)		FIC index	Interaction mode
			AgNPs + strep.			
*B. cereus*	15.625	5.85	3.90	0.73	0.374 ± 0.12	Synergistic
*S. aureus*	18.75	8.59	4.68	1.07	0.374 ± 0.08	Synergistic
*E. coli*	18.75	8.59	4.68	0.53	0.311 ± 0.04	Synergistic
*K. pneumoniae*	15.625	5.85	3.90	0.73	0.374 ± 0.11	Synergistic

**Table 4:** Minimum inhibitory concentration (MIC) against pathogenic fungus alone and in combination with antifungal (fluconazol) along with interaction index. Experimentswere performed in triplicates; mean ± SD are shown

MIC (μg)			Interaction index			
Fungi	AgNPs	Fluconazole	FIC (μg)		FIC index	Interaction mode
			AgNPs + fluconazole			
*C. albicans*	14.06	7.81	3.51	0.68	0.336 ± 0.06	Synergistic
*C. glabrata*	14.06	5.4	3.51	0.34	0.312 ± 0.06	Synergistic

#### 3.3.3. *In vitro* antioxidant activities of capped AgNPs

Cellular respiration leads to the production of reactive oxygen species (ROS), reactive nitrogen species (RNS) and various other kinds of free radicals possessing unpaired valence shell electrons. These notorious molecules play vital role in cell signaling but also when in excess leads to oxidative damage to the cell by reacting with the cellular components that cause cancer, aging, cataract, cardiovascular diseases, dysfunction of organs. Thus, antioxidants play a crucial role to down regulate and eliminate free radicals before they damage the cell.^47^ Five different assays (PM, FRAP, DPPH, ABTS and NOR) were opted to measure antioxidant properties of capped AgNPs, aqueous cell extract (ACE) and standard ascorbic acid (AA) (Table 5 & Fig. S5†). The concentrations at which 50% scavenging (IC_50_) of free radicals were calculated.

**Table 5:** Antioxidants properties of (PM, ABTS, FRAP, DPPH and NOR assay) of capped AgNPs

Antioxidant activity (IC_50_)	Aqueous cell extract (ACE) (μg mL^−1^)	Capped AgNPs (μg mL^−1^)	Ascorbic acid (AA) (μg mL^−1^)
PM	154.78 ± 2.13	87.20 ± 1.53	5.87 ± 0.023
ABTS	169.84 ± 2.53	42.87 ± 0.18	12 ± 0.05
FRAP (EC_1_)	324.5 ± 4.53	203 ± 2.42	9.29 ± 0.058
DPPH	176.03 ± 3.67	52.04 ± 1.45	6.24 ± 0.72
NOR	170.57 ± 4.15	54.04 ± 1.23	6.77 ± 0.03

For total antioxidant activity, phospho-molybdenum assay (PM) was adopted which depends on the reduction of phosphate-molybdenum(vi) to phosphate molybdenum(v) by antioxidants. The incubation of the sample with molybdenum(vi) determined the presence of antioxidants in the sample, which was assessed by measuring the absorbance of reduced green molybdenum complex.^48^ The results revealed that capped AgNPs and ACE and standard AA showed maximum antioxidant potential at concentration 175 μg mL^−1^ (81.22% ± 0.013), 300 μg mL^−1^ (74.91% ± 0.135) and 14 μg mL^−1^ (93.26% ± 0.05) respectively. IC_50_ of the AgNPs, ACE and AA were found to be 87.20 ± 1.53, 154.78 ± 2.13 μg mL^−1^ and 5.87 ± 0.023 respectively (Table 5). Dhayalan *et al.* (2017) determined total antioxidant activity of AgNPs derived from *Embelia ribes* and found IC_50_ at 60 μg mL^−1^ for the same assay.^49^

ABTS˙^+^ is a pre generated free radical and the interaction between antioxidant and ABTS˙^+^ causes bleaching of ABTS˙^+^.^50^ Steady inhibition of ABTS˙^+^ free radical (14.06% ± 0.005 to 75.33% ± 0.0001) was observed in the concentration ranging from 10 to 90 μg mL^−1^ of capped AgNPs. The IC_50_ for capped AgNPs, ACE and standard AA were found to be at 42.87 ± 0.18, 169.84 ± 2.53 and 12 ± 0.05 μg mL^−1^ respectively (Table 5). Moteriya and Chanda (2017), also reported 57% inhibition of ABTS˙^+^ free radicals with 60 μg mL^−1^, AgNPs synthesized by using *Caesalpinia pulcherrima* flower extract.^51^

In FRAP assay of capped AgNPs and ACE reducing capacity measured by their ability to reduce ferric tripyridyltriazine (Fe^3+^-TPTZ) to ferrous tripyridyltriazine (Fe^2+^-TPTZ) ended up with a formation of blue color complex which is proportional to the amount of antioxidant.^52^ In the present study, increased absorbance with increasing concentration of samples and results were expressed in terms of equivalent concentration (EC1) by plotting regression curve for ACE (*y* = 0.0028*x* − 0.0914, *R*^2^ = 0.9578), capped AgNPs (*y* = 0.0049*x* + 0.0053, *R*^2^ = 0.955) and AA (*y* = 0.0982*x* + 0.0871, *R*^2^ = 0.9749) with the reference of ferrous sulfate (*y* = 0.002*x* + 0.0741, *R*^2^ = 0.9863). ACE, AgNPs and AA showed EC_1_ values at 324.5 ± 4.53, 203 ± 2.42 and 9.29 ± 0.058 μg mL^−1^ respectively (Table 5). FRAP activity of green synthesized AgNPs is also reported by Nayak *et al.*, (2016).^53^

DPPH radical scavenging activity at concentration range 10–80 μg mL^−1^ showed scavenging percentage ranging from 6.23% ± 0.008 to 73.76% ± 0.004 which directly depends on hydrogen donating tendency of sample to DPPH radical. IC_50_ value of capped AgNPs, ACE and standard AA were observed at and 52.04 ± 1.45, 176.03 ± 3.67 and 6.24 ± 0.72 μg mL^−1^ respectively (Table 5). In previous reports, *Trichodesmium erythraeum* and *Ecklonia cava* derived AgNPs showed 37.15 and 50% scavenging percentage at 100 and 198 μg mL^−1^ respectively against DPPH with increasing concentration.^54,55^

Nitric oxide radical scavenging assay was also performed for antioxidant analysis. Nitric oxide (NO) is an important bioregulatory molecule in the nervous, immune and cardiovascular systems. Many progressive diseases including atherosclerosis, hypertension and neuro-degeneration are associated with NO derived oxidants.^56^ Sodium nitroprusside decomposes in aqueous solution at pH 7.2 and produces NO·, this NO· then reacts with oxygen to produce nitrite and nitrate which are quantified by Griess reagent.^57^ Samples showed a concentration dependent scavenging activity ranging between 22.20 ± 0.48 to 91.36% ± 0.25 at concentration range 25–175 μg mL^−1^ for capped AgNPs and 16.74 ± 1.27 to 77.47% ± 0.23 for ACE in the concentration range from 25 to 300 μg mL^−1^. IC_50_ of capped AgNPs, ACE and AA were found to be 54.04 ± 1.23, 170.57 ± 4.15 and 6.77 ± 0.03 μg mL^−1^ respectively (Table 5). AgNPs synthesized by microalgae *Trichodesmium erythraeum* showed 88.12 ± 0.26% scavenging activity at 500 μg mL^−1^.^58^ AgNPs synthesized by seaweeds *Sargassum wightii* and *Ecklonia cava* aqueous extract possess strong antioxidant activity due to presence of phenols.^55,58^

#### 3.3.4. Anti-inflammatory activity of capped AgNPs

**Fig. 7:**
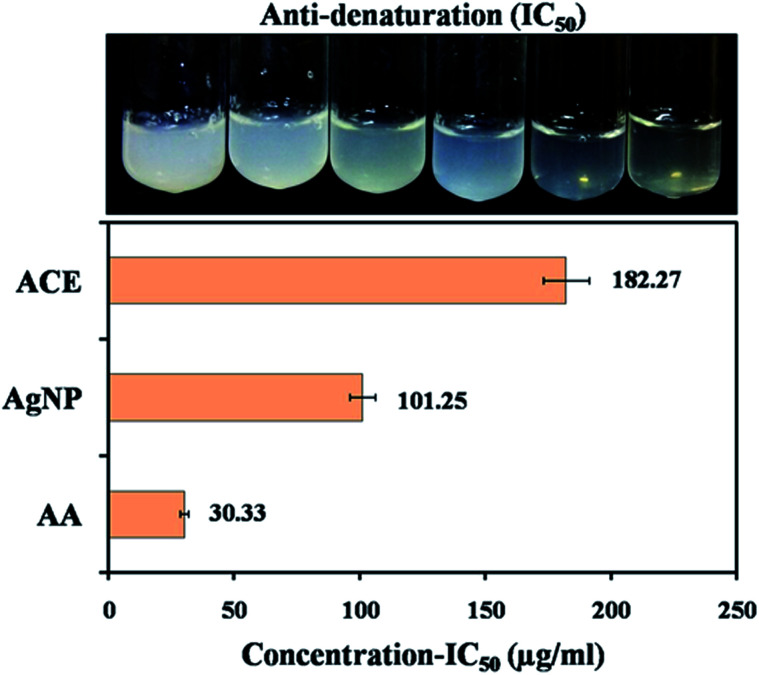
(A and B) Anti-denaturation activity of protein against capped AgNPs as anti-inflammatory agent.

Inflammation is a complex process, which is frequently associated with pain and involves occurrences such as, the increase of vascular permeability, increase of protein denaturation and membrane alteration. During denaturation, proteins lose their tertiary and secondary structure by application of stress or heat which causes inflammation. Maximum inhibition of protein denaturation observed was 72.07% ± 0.45 with 175 μg mL^−1^ AgNPs and 64.83% ± 0.91 with 300 μg mL^−1^ AgNPs. Aspirin (acetylsalicylic acid), a standard inflammatory drug showed maximum inhibition 93.21% ± 0.27 at the concentration of 100 μg mL^−1^. IC_50_ of acetylsalicylic acid, AgNPs and cell extract was found to be 30.33 ± 0.23, 101.25 ± 1.54 and 182.27 ± 3.76 μg mL^−1^ respectively (Fig. 7B). Bouhlali *et al.*, 2020 also observed similar results which can be due to the combined effect of bioactive agent adsorbed over AgNPs surface enhancing their dispersibility and bioavailability.^59^

### 3.4. Biocompatibility assay of capped AgNPs

**Fig. 8:**
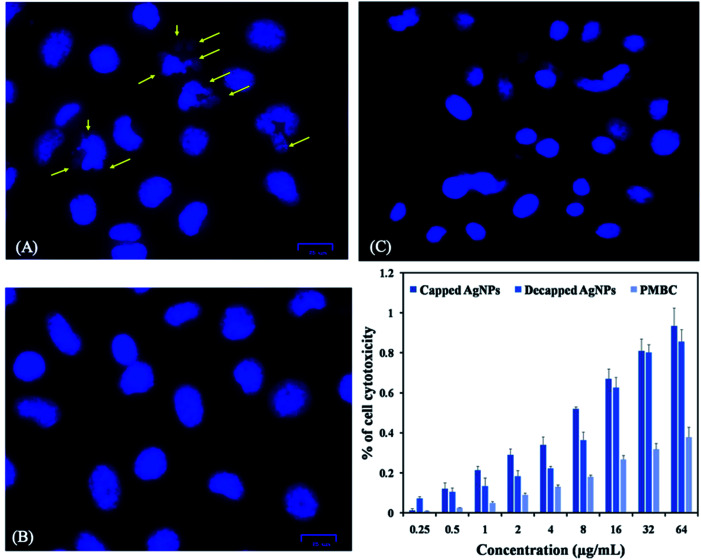
Experimental observation of the cytotoxic property of *Plectonema*-derived AgNPs: (A) cytotoxic activity of capped AgNPs at IC_50_ concentration against lung cancer cell line (A549); (B) A549 control; (C) cytotoxic activity (IC_50_) of capped AgNPs against normal (peripheral mononuclear blood cells) PMBC cells; (D) cytotoxic activity of capped, decapped AgNPs against A549 lungs cancer cell line and normal PMBC cells at different concentration. Experiments were performed in triplicates; the error bar represents statistically significant differences (*p*-value < 0.05).

Peripheral Blood Mononuclear Cell (PBMC) was taken as normal mammalian cells. AgNPs exhibited IC_50_ values for PBMC was >35 μg mL^−1^, this showed intact nuclei of uniform shape and size with smooth edges, which indicated that normal cells were almost unaffected by capped AgNPs (Fig. 8C). Capped AgNPs exhibited low haemolytic activity may be useful in administration of some medical devices. The AgNPs toxicity of PMBC might be due to free silver ions release, total silver ion concentration or interaction between cellular components and nanoparticles.^60^ Production of silver ions, decomposition, binding as well as membrane vesiculation may be the mechanisms responsible for induction of hemolysis.^61^ AgNPs synthesized from cyanobacterium *Oscillatoria limnetica* showed anti-hemolytic activity of AgNPs was being non-toxic to human RBCs at low concentrations.^40^

**Fig. 9:**
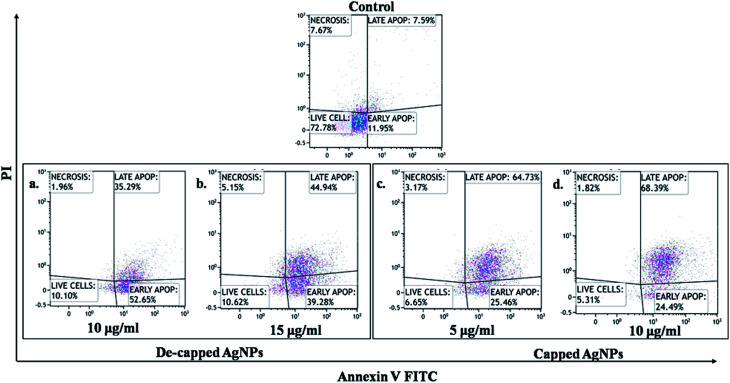
Apoptosis analysis against lung cancer cell line (A549) cells determined by APC Annexin V/FITC apoptosis detection assay exposed to AgNPs.

In the present study we also investigated effect of capped and de-capped AgNPs against non-small cell lung cancer (A549) cell line. Our study showed that treatment with AgNPs *in vitro* reduced A549 cancer cell line viability in a dose-dependent manner. IC_50_ values calculated for capped AgNPs was 5.53 μg mL^−1^ (*y* = 0.067*x* + 0.129; *R*^2^ = 0.985) where as for decapped AgNPs was 12.39 μg mL^−1^ (*y* = 0.033*x* + 0.091; *R*^2^ = 0.993) (Fig. 8D) *In vitro* AgNPs treatment induced apoptosis in the cell line as visualized by DAPI staining, Fig. 8A and B showed nuclei blabbing, condensation and cracking and these are the characteristic features of apoptosis. Further Annexin-FITC/PI assay using phosphatidylserine staining of non-small cell lung cancer (A549) cells was performed to find out quantitative changes that occur during apoptosis in response to capped and de-capped AgNPs treatment. Significant increase in phosphatidylserine at the surface of A549 cells with an increase in AgNPs exposure was showed. Fig. 9 shows that untreated cells of A549 did not showed significant apoptosis, whereas capped AgNPs with 5 μg mL^−1^ & 10 μg mL^−1^ and decapped AgNPs with 10 μg mL^−1^ & 15 μg mL^−1^ treated cancer cells become apoptotic after 48 h with early apoptotic cells population of 25.46% & 24.49% for capped AgNPs and 52.65% & 39.28% for decapped AgNPs along with apoptotic population against capped AgNPs was 64.73% & 68.39% and against decapped AgNPs was 35.29% & 44.94%. Changes in the viable cell population mean that the AgNPs showed significant antitumor activities. Similarly Sanpui *et al.* (2011) demonstrated that the chitosan mediated AgNPs, disrupt the normal cellular function, and also affect its membrane integrity by inducing apoptotic signalling genes of mammalian cells that causes death.^62^ According to the recent research AgNPs have been proven to induce cytotoxic effect *via* autophagy, mitochondrial dysfunction, arrest of the cell cycle, and causing lipid peroxidation also lead to generation of reactive oxygen species producing apoptosis.^63–66^

## 4. Conclusion

In the quest for advanced topical ointments, wound healing bandages and coated stents with superior resistance to microbial infections, nanosilver formulations have surfaced as an attractive option. To the best of our knowledge, the present investigation demonstrated for the first time that the presence of capping molecules significantly influences the activity of biologically synthesized nanoparticles using cyanobacteria. Our characterization marks the crystalline nature of the particles, spherical in shape with moderately dispersed nanoparticles as well as presence of intrinsic capping and stabilizing protein on the surface of *Plectonema* NCCU-204-derived AgNPs. Removal of capping molecules (protein shell) from the surface of AgNPs showed significant decrease in anti-bacterial activity against both Gram-positive and Gram-negative bacteria. Furthermore, these AgNPs combined with the antibiotics and fungicides exhibited a significant synergistic effect. The facile synthesis and salient features of this AgNPs with biocompatibility with potential cytotoxicity against cancer cell line facilitate their potential applications to the scientific foundation for translational studies in animal that may also solve problem associated with microbial multidrug resistance.

## Author's contribution

AZ and NA are joint first co-authors, NA, AZ, and TF: conceived and designed the experiments; NA, AZ, RA, IA, DY performed the experiments; AC, VH, and MSA helped in flow cytometry. NA, AZ, TF, PS, RK, SMA Analyzed the data; BA helped in the preparation of ESI.† NA and AZ prepared the draft; TF and NA proofread the final draft. All authors approved the final manuscript.

## Conflicts of interest

There are no conflicts of interest to declare.

## Supplementary Material

RA-012-D1RA08396A-s001
