# Supplementary material for: Synthesis, purification and characterization of Plectonema derived AgNPs with elucidation of the role of protein in nanoparticle stabilization
Source: RSC Adv. 2022 Jan 18;12(4):2497–510. doi: 10.1039/d1ra08396a (PMC8979216; doi:10.1039/d1ra08396a)
Supplement: RA-012-D1RA08396A-s001 [file RA-012-D1RA08396A-s001.pdf]

Electronic Supplementary Material (ESI) for RSC Advances.
This journal is © The Royal Society of Chemistry 2022

S1. Cyanobacterial screening for the synthesis of silver nanoparticles

For biosynthesis of AgNPs, protocol of Aziz et al. was adopted with slight modifications (Aziz et al. 2015). All cultures were harvested in the mid-exponential phase, transferred to 50 mL conical centrifuge tubes (Himedia, India) and centrifuged at 6000 rpm for 5 min at 4 ℃. *Spirulina* and *Arthospira*sp. were collected by filtration. The pellets were washed thrice with double distilled water (ddH_2O) to remove traces of media by repeating centrifugation process. Thus obtained wet cyanobacterial biomass (5 g) was added to 15 mL ddH_2O and homogenized for 5 min then sonicate for 150 s using vibra cell probe sonicator (10:10 s on and off cycle at 25% Amp) followed by incubation at 100 ℃ in water bath for 10 min in 100 mL Erlenmeyer flask, and then subsequently cooled at room temperature. It was further centrifuged at 7000 rpm for 10 min, the obtained supernatant was filtered using Whatman filter paper no. 1, thus obtained soup was called as aqueous cell extract (ACE). Biosynthesis of AgNPs was carried out by adding 10 mL ACE to 90 mL $AgNO_3$ (1 mM) solution, followed by incubation at 30 ± 1 ℃ for 24 h under 2000± 100 lux in the incubator (MRC orbital shaker, Israel). Bioreduction of $AgNO_3$ to AgNPs was monitored using a double beam UV−VIS spectrophotometer (UV-1800, Shimadzu, Japan) at 24 h time intervals. Specifically, the wavelength range 300−700 nm was used to assess the change in optical properties**.** To collect the nanoparticles the reaction mixture was centrifuged at 4000 rpm for 5 min then supernatant was taken and centrifuged at 15000 rpm for 20 min. The resultant pellet was collected, re-dispersed in ddH_2O and then centrifuged. The above procedure was performed thrice to ensure complete removal of extraneous residue followed by lyophilization to get powdered nanoparticles.

Thereafter, scanning electron microscopy (Hitachi S-4500 SEM) was performed to collect the data of surface morphology (shape and size). For SEM imaging, 1 mg of silver nanoparticles was added in 5 ml of ddH_2O followed by sonication for 1 min and 10 μL of it was placed over copper grid and left it to air dry. The sample was then coated with gold in a sputter coater (Quorum) before measurement and the acquired images were analyzed using ImageJ (http://imagej.nih.gov/ij/download.html). Based on reaction time and size of silver nanoparticles we have selected the strains that had the maximum potential to reduce $AgNO_3$ to AgNPs for further production and studies.

As expected cyanobacterial extract mixed with 1mM $AgNO_3$ solution leads to color transition from greenish to yellowish brown indicating formation of silver nanoparticles via reduction of Ag^+ ions to elemental form (Ag^o) in the presence of enzymes reducing agents such as NADPH dependent nitrate reductase present inside the cell extract (Jena et al. 2013). In most of the strains, reaction started within 2 h. AgNPs shows spectral peak(s) in between 410 and 460 nm (**Figure S1. A-E**) occurs due to Surface

Plasmon Resonance (SPR) which happens due to resonance of free electron vibration (Mukherjee et al. 2001). According to Patel et al. (2015), extracellular polysaccharide, pigment phycocyanin, and some other biomolecules act as capping agents on the surface of nanoparticles during synthesis. Bio-fabrication of AgNPs using *Spirulina Platensis* and *Nostoc linckia* reported by Cepoi et. al. 2014, suggested that synthesis mainly occur due to presence of abundant number of bioactive compounds and metabolites. In addition, Sathish kumar*et al.* 2019suggested the role of secondary metabolites like saponins, quinines, flavonoids and terpenoids in the biofabrication of silver nanoparticles. Ali et al. 2011b, used extract of *Oscillatoria Willei NTDM01* to synthesize silver nanoparticles and suggested the involvement of proteins as a capping molecule for its stabilization. In this study all the selected strains showed positive results for synthesis of silver nanoparticles with variations in reaction time, size and shape depicted in **Table S1**. Most of the strains synthesized spherical nanoparticles where as three strains synthesized cubic and only one produced pentagonal nanoparticle. The minimum reduction at which the synthesis starts was 30 min for *Plectonema sp. NCCU 204* and *Haplosiphon fontinalis* NCCU-339 took maximum reduction time (270 min) for the synthesis of nanoparticles. So we observed that *Plectonema sp. NCCU 204* stood out with least reduction time (30 min), smallest average size range (9-17 nm) with spherical in shape and thus selected as the best strain for nanoparticles synthesis and used for further studies (**Figure 1S. A-E**). Similar observation was also observed by Ahamad et al., 2021 while working with *Anabaena variabilis* the minimum reduction time was 1 h at absorption peak at 440 nm with an average size range of 11-15 nm with TEM.

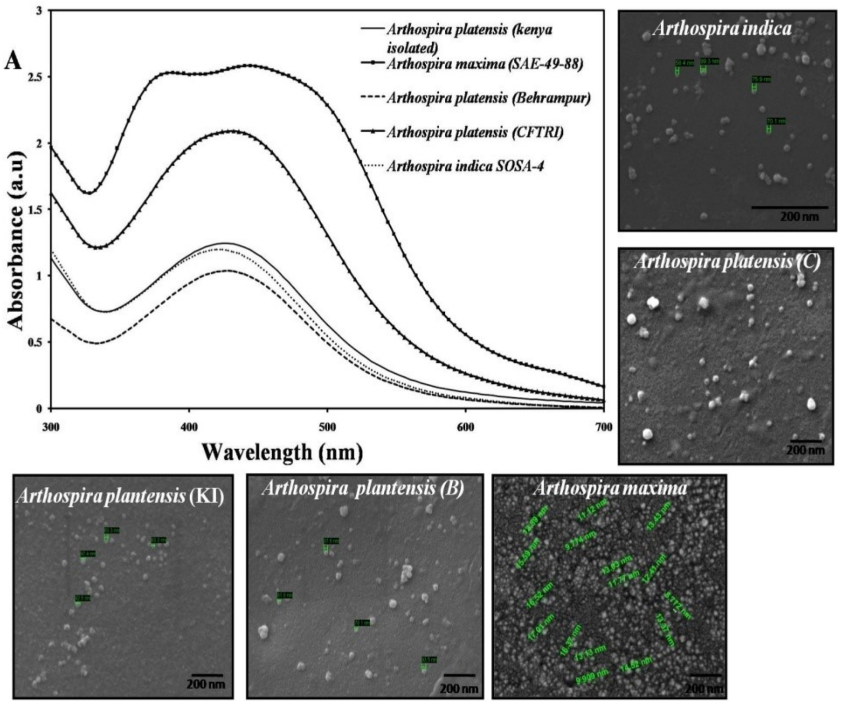

Figure S1 (A): SEM micrograph of the selected cyanobacteria with UV-VIS spectrophotometer

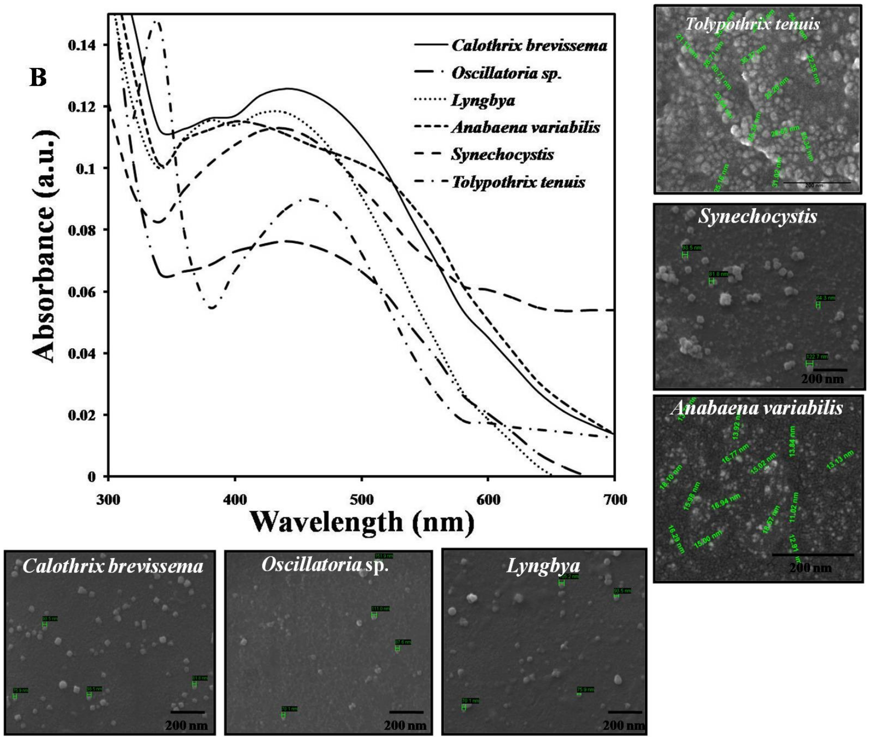

Figure S1 (B): SEM micrograph of the selected cyanobacteria with UV-VIS spectrophotometer

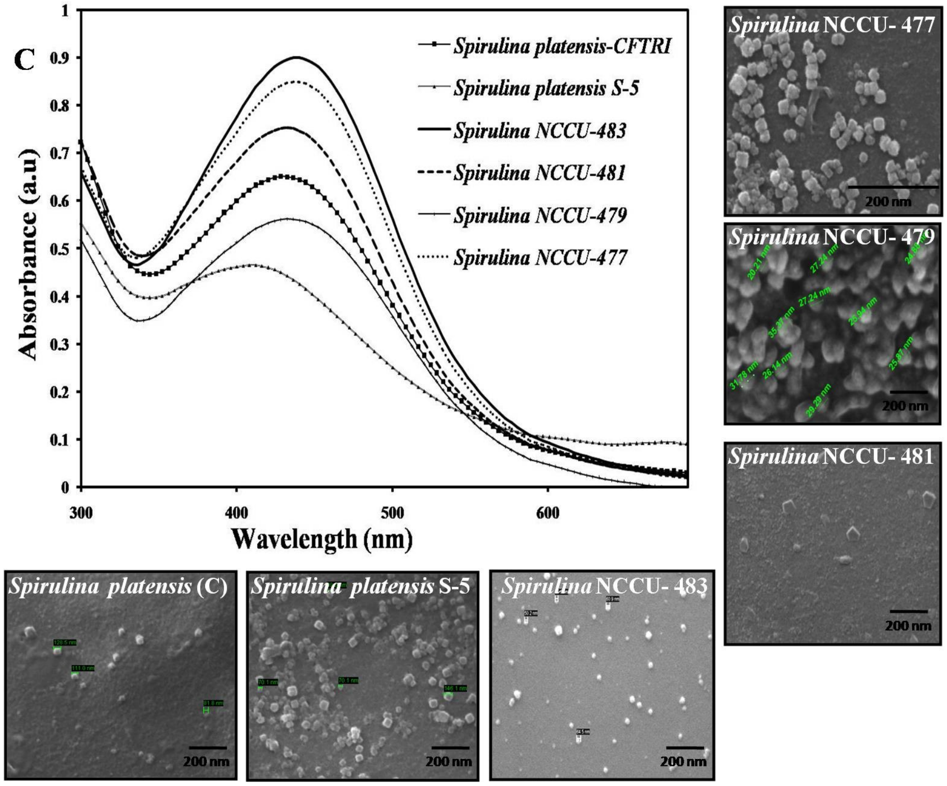

Figure S1 (C): SEM micrograph of the selected cyanobacteria with UV-VIS spectrophotometer

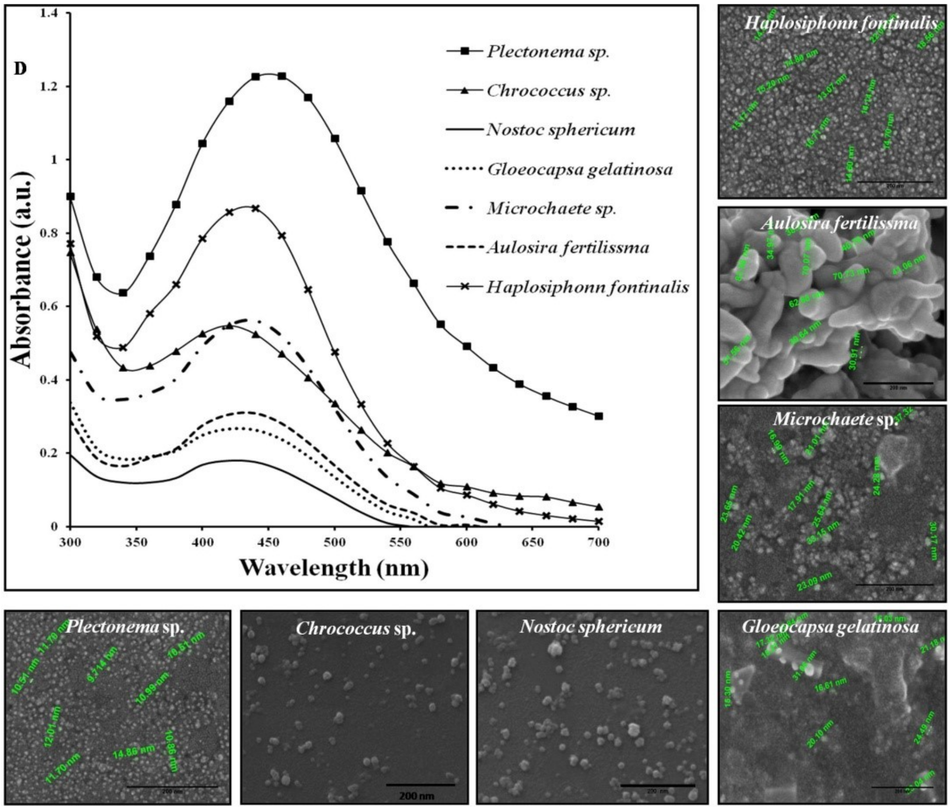

Figure S1 (D): SEM micrograph of the selected cyanobacteria with UV-VIS spectrophotometer

E

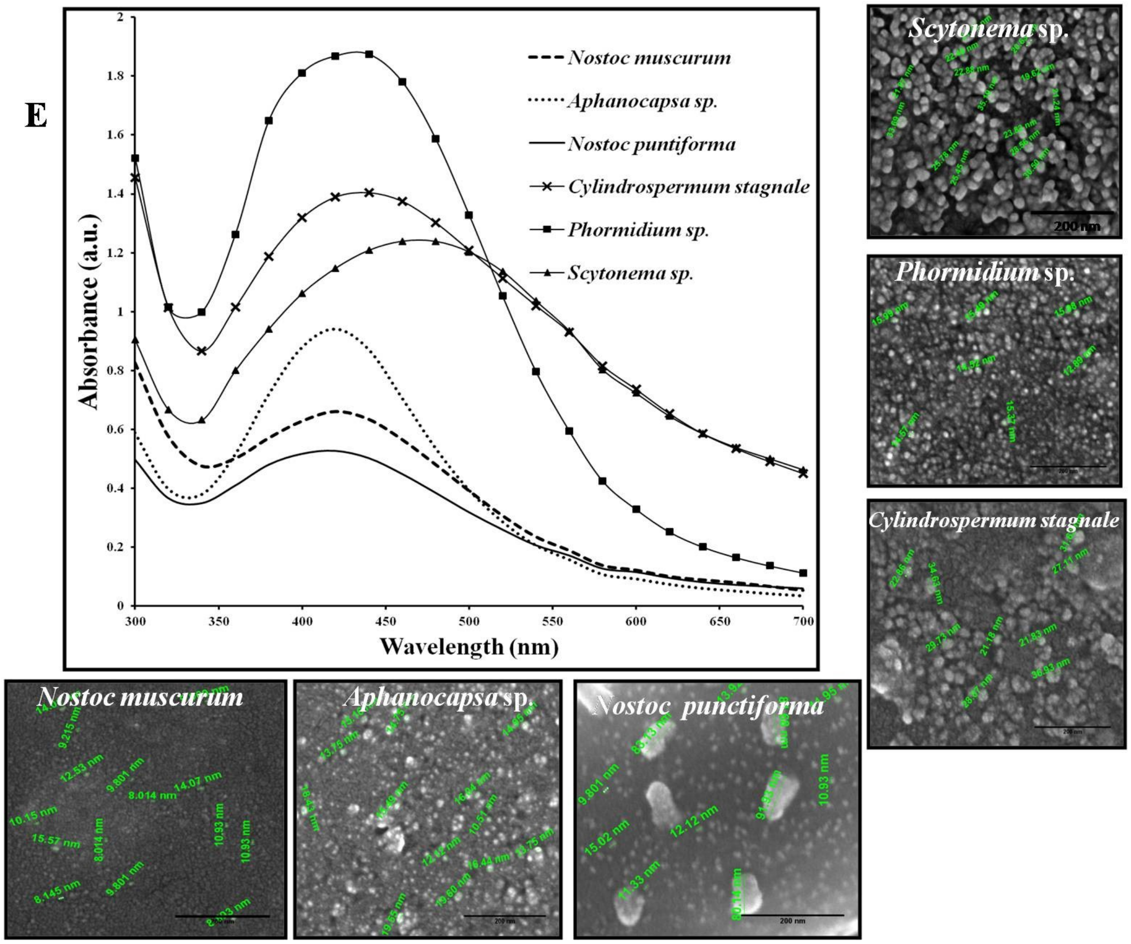

Figure S1 (E): SEM micrograph of the selected cyanobacteria with UV-VIS spectrophotometer

Table S1. Range of size of silver nanoparticles obtained by SEM image analysis

S. No.	Cyanobacterial species	Minimum Reaction Time (Min)	Shape of Particles	Maximum absorption peak	Range of particles size (nm)
1	***Plectonema*sp. NCCU-204**	**30**	Spherical	420	**9-17**
2	*Arthrospira maxima* SAE-49-88	35	Spherical	440	10-25
3	*Arthrospira platensis* (Behrampur)	35	Spherical	428	30- 90
4	*Arthrospira platensis* (CFTRI)	46	Spherical	432	30- 70
5	*Arthrospira indica* SOSA-4	60	Spherical	420	20- 70
6	*Calothrix brevissema* NCCU-65	200	Cubic	440	30- 85
7	*Oscillatoria* sp. NCCU-369	150	Spherical	440	35- 80
8	*Lyngbya* NCCU-102	120	Spherical	432	25- 60
9	*Anabaena variabilis* NCCU-441	52	Spherical	440	11-25
10	*Synechocystis* NCCU-370	52	spherical	432	15- 45
11	*Spirulina* NCCU-483	65	Pentagonal	440	35- 90
12	*Spirulina* NCCU-477	45	Cubic	436	30-90
13	*Spirulina platensis* NCCU-S5	45	Cubic	412	20- 40

14	*Spirulina* NCCU-479	45	Spherical	432	11-35
15	*Spirulina* NCCU-481	45	Spherical	432	10-30
16	*Arthrospira platensis* (Kenya isolated)	45	Spherical	424	50-95
17	*Chrococcus*sp. NCCU-207	120	Spherical	424	20- 70
18	*Nostoc sphericum*	220	Spherical	433	40-80
19	*Gloeocapsa gelatinosa*NCCU-430	45	Spherical	430	15- 40
20	*Microchaetesp.*NCCU-324	40	Spherical	434	15- 40
21	*Aulosira fertilissma*NCCU-443	58	Spherical	428	30-75
22	*Haplosiphonn fontinalis* NCCU-339	270	Spherical	427	13- 40
23	*Nostoc muscurum*NCCU-442	250	Spherical	420	9- 30
24	*Aphanocapsa*	80	Spherical	418	12- 40
25	*Nostoc punctiforma*	170	Spherical	418	10-50
26	*Cylindrospermumstagnale*	75	Spherical	440	15-46
27	*Phormidium*sp. NCCU-104	50	Spherical	428	12- 25
28	*Scytonema*sp.	170	Spherical	465	18- 30
29	*Tolypothrix tenuis* NCCU-122	180	Spherical	440	20- 40
30	*Spirulina platensis* CFTRI	46	Spherical	428	15-35

S2. Optimization of nanoparticles synthesis by the most potent strain

Best selected strain *Plectonema*NCCU-204was further optimized for AgNPs synthesis and was harvested at mid exponential phase, in which the biomolecules are in the stage of optimal activity leading to maximum reduction of $AgNO_3$ for AgNPs. Extract efficiency was analyzed at different temperatures and time, the biomass was homogenized and sonicated (water bath sonicator) for 10 min and kept it for different temperatures (60, 80 and 100 ℃) in water bath for 10 min and 20 min and the synthesized AgNPs were subjected to UV-VIS spectrophotometer to study the effect of time and temperature on the rate of synthesis of silver nanoparticles. Biomass was exposed to 100℃ for 10 min in water bath gave best results to produce AgNPs (**Figure S2A**). Below 100 ℃, synthesis of AgNPs was very slow while exposure at 100℃ for longer time (20 min) shows no reduction up to 5 days.

The reaction mixture ($AgNO_3$ + Aqueous cell extract) was incubated at different temperatures (20, 30, 40, 50 °C) to obtain the effective reaction temperature at which maximum reduction of $AgNO_3$ can be obtained while other parameters were kept fixed (100 ℃ for 10 min extract efficiency, 1mM $AgNO_3$ concentration and 7.4 reaction pH). The effective reaction temperature at which maximum reduction of $AgNO_3$ to AgNPs synthesis was observed to be at 30 ºC (**Figure S2B**). At lower temperature (< 20 ºC) reduction process starts after 10 days (data not shown) while at higher temperature >40 ºC rate of reaction is faster but quick aggregation of AgNPs was observed. As the temperature increase there was red shift with broadened absorption peak leads to aggregation and increase in the size of nanoparticles. Elevation in temperature can increase the reaction kinetics (Brownian movement) thus enhancing

aggregative properties of nanoparticles (Piñero et al. 2017). Hamida et al. 2020 also observed that silver nanoparticles can be synthesized at room temperature with an average size of 31 nm.

Then the reaction mixture was incubated at different concentration of $AgNO_3$ (1mM, 3mM, 5mM and 7mM), to obtain the effective reaction concentration of $AgNO_3$ at which maximum reduction of $AgNO_3$ can be obtained while other parameters were kept fixed (100 ℃ for 10 min extract efficiency, 30 ℃ reaction temperatures and 7.4 reactions pH). As we increase the concentration of the salt ($AgNO_3$) the reduction potential increases but SPR decreases. According to Brause et al. 2002, shifting of peak towards the UV-regions (Blue shift) indicating decreased the particles size, but it shifts towards visible region (red shift) if particles size increases. In our study, the effective concentration for large scale production of AgNPs was found to be at 5mM, but large, uncontrolled and aggregated particles were also observed. While at 1mM concentration, SPR is more towards UV-VIS region with no aggregation of nanoparticles (**Figure S2C**). Such observations were also noticed by earlier workers with cyanobacteria (Husain et al. 2015).

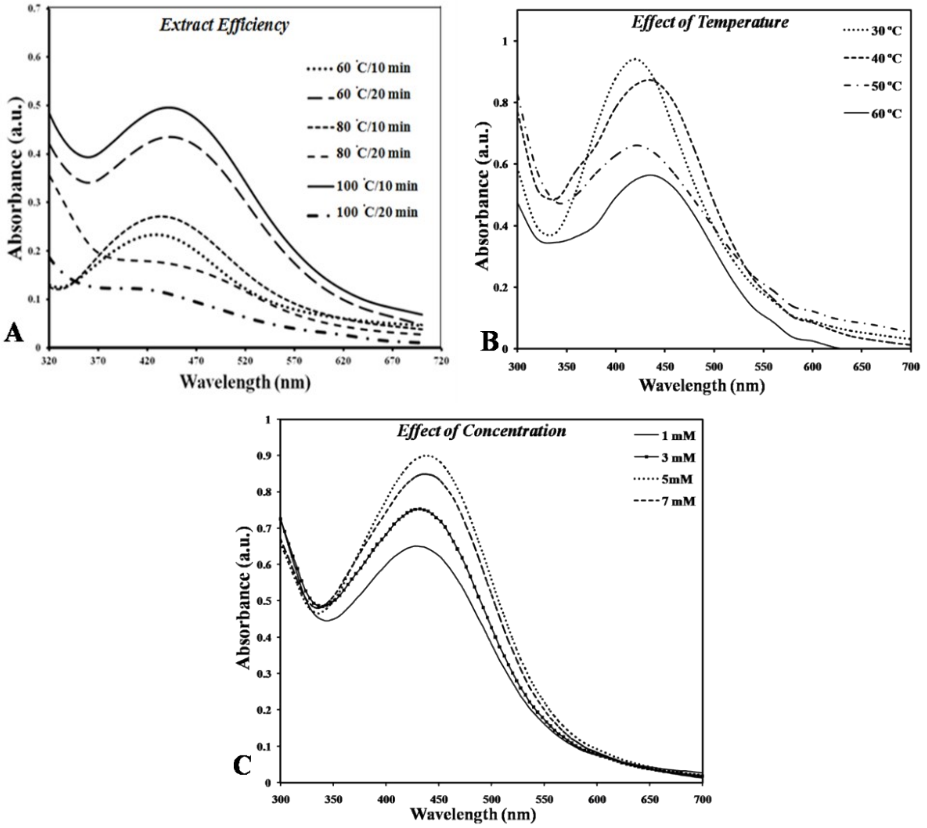

Figure S2 (A). Extract efficiency at different time interval and temperature; **(B).** Effect of reaction temperature for production of AgNPs; **(C).** Effect of concentration for production of AgNPs,

One of the key experimental parameters in biogenic nanoparticles synthesis is the pH of the reaction medium. The reaction mixture for nanoparticles synthesis were kept under a range of pH conditions (4.4 - 8.4 pH) to see the effect on the synthesis ability while time and temperature for aqueous extract preparation (10 min at 100℃), reaction temperature (30℃) and $AgNO_3$ concentration (1 mM) were kept fixed. Indeed, a salient feature of biological synthesis is their ability to operate under a range of pH conditions (4.4, 5.4, 6.4, 7.4, 8.4 pH) with the production of nanoparticles of different morphologies. Here, the maximum absorbance under acidic conditions (pH 4.4) was found to occur at 420 nm (**Figure S2.1 A**) whereas, in a more alkaline environment (pH 8.4) at 460 nm suggested red shift (**Figure S2.1 E**). In the acidic condition the peak becomes broader and the size of particle increases. In alkaline conditions (pH > 7) hydroxide ion act as strong reducing agent and showing additive effect with proteins present in the extract that enhance the reduction of metallic ions by several folds (Phanjom and Ahmed 2017). At pH 7.4 synthesis of nanoparticles was fast with no aggregation and graph is more symmetrical **(Figure. S2.1D)**. Krishnaraj et al. (2012), also reported near to neutral pH as an ideal condition for AgNPs synthesis and its stabilizations. Hence, in our study, we presume that slightly alkaline condition is favorable for controlling the particle size.

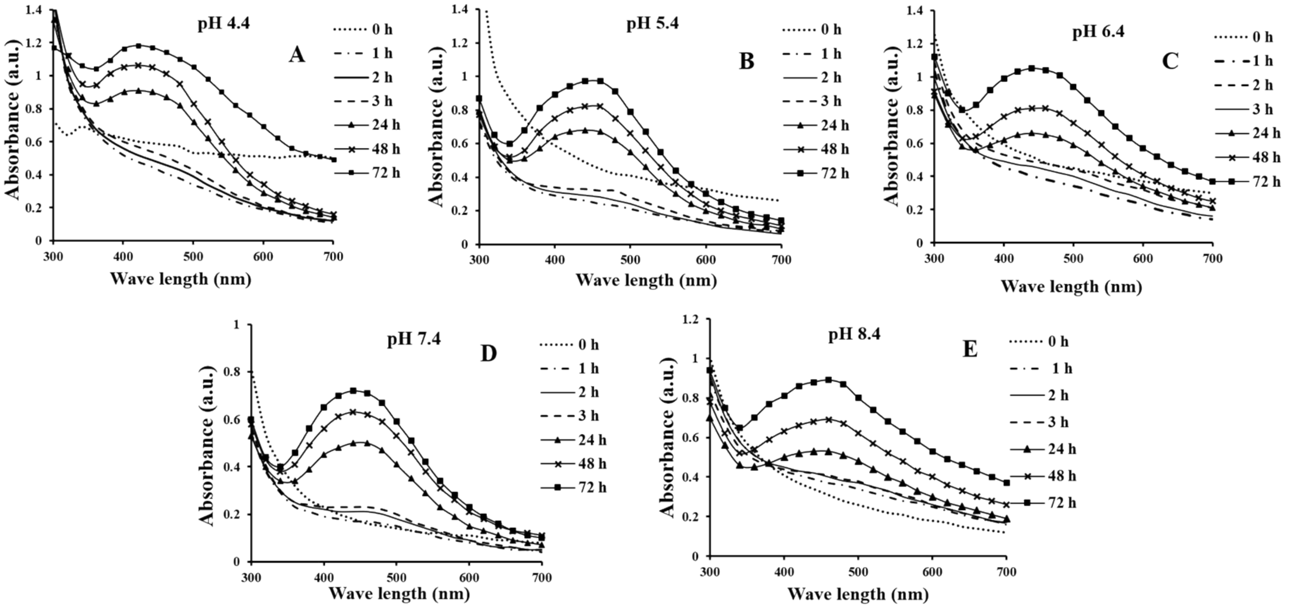

Figure S2.1 D. UV-Vis spectra of synthesized AgNPs recorded at different pH

Using all optimized conditions (*Plectonema NCCU-204* aqueous extracts preparation at 100℃ for 10 min, 1 mM $AgNO_3$ solution, pH 7.4 and incubation temperature 30 ºC) taken to synthesize silver

nanoparticles (AgNPs). Expectedly, the nanoparticles were synthesized when the component of extract reduce the Ag^{+} ions into their corresponding elemental form through the enzymes generated by the cellular activities (Aziz et al. 2019). During the reduction process (from $AgNO_3$ into Ag), we observed a gradual change in the color of the solution from colorless to yellowish brown over a 72-h period (**Figure 1**) indicating the rapid synthesis and stabilization of biosynthesized AgNPs. The surface plasmon resonance (SPR) was found to increase at 440 nm at different time interval indicating the synthesis of AgNPs. It was noted that the reduction of $AgNO_3$ solution into AgNPs started within 1 h after the addition of $AgNO_3$ solution into cell extract and completed at 72 h after that reaction saturation was observed. Maximum absorption peak of *Chlorella pyrenoidosa*derived silver nanoparticles was observed after 72 hours of incubation of reaction mixture (Aziz et al. 2015).

Finally, silver nanoparticles were synthesized by considering all the optimized conditions. The purification of obtained nanoparticles was done through washing (ddH_2O), organic solvents (acetone/ethanol), dilution, centrifugation.

S3. Antibacterial activity of water and ethanol washed capped AgNPs

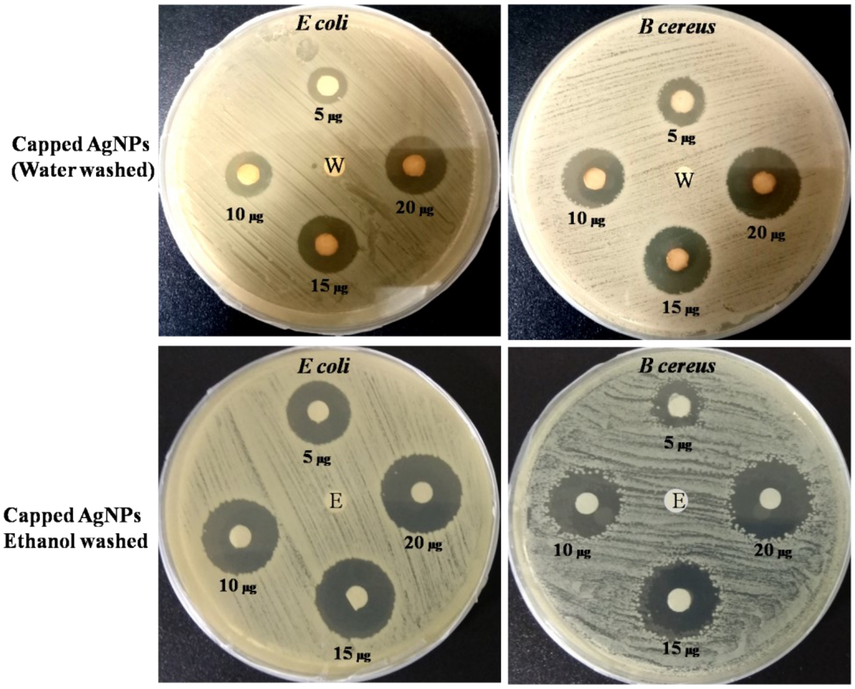

Figure S3: Antibacterial activity of capped AgNPs

S4. Antimicrobial activity of capped AgNPs

To assess the anti-bacterial efficacy of biologically synthesized AgNPs, a disc diffusion method or Kirby-Bauer method (Bauer et al. 1966) was performed against both gram negative bacteria (*Escherichia coli, Klebsiella pneumonia*) as well as gram positive bacterium (*Bacillus subtilis*, *Staphylococcus aureus*) on Mueller-Hinton agar plates.Antifungal activity was also determined against *Candida albicans* and *Candida glabrata* following standard guidelines of CLSI (CLSI 2008). To develop the inoculums of each bacterium, single colony was transferred to separate test tubes containing sterile Muller–Hinton liquid medium and incubated overnight at 37 ℃ on a shaker incubator at 150 rpm to obtain pure and fresh culture. Overnight grown culture was diluted to 0.5 McFarland standards with sterile media. Then 100 µL of it was transferred onto Muller–Hinton agar plates and bacterial lawn was made using sterile swabs, different masses of 5, 10, 15, and 20µg of Ag NPs were loaded from the stock solution of 1mg/mL^{-1} were added of 6 mm size disc.Antifungal activity was also determined by disc diffusion method against *Candida albicans* and *Candida glabrata* following standard guidelines of CLSI (CLSI 2008). Strains were grown overnight at 37 ℃ on incubator shaker at 150 rpm into YPD (1:2:2) broth medium. Approximately 10^{5}cells/mL were inoculated into molten YPD agar media and poured into petriplates. Different amount of AgNPs (5, 10, 15, 20 and 25µg) were pipetted onto 6 mm sterile filter discs. Fluconazole 10 µgfrom the stock solution of 1mg/mL^{-1}was taken as positive control. Plates were labeled and incubated at 37 ℃ for 16 h. Zones of inhibition were recorded in millimeters to express antimicrobial activity. Experimental work was done in triplicates and mean value was taken as a result.

Cyanobacterial mediated synthesis of AgNPs exhibited effective antibacterial activity when tested against both Gram positive bacteria (*Staphylococcus aureus*, *Bacillus cereus*) and Gramnegative bacteria(*Escherichia coli* and *Klebsiella pneumonia*). Highest zone of inhibition was observed against Gram negative *Klebsiella pneumoniae* (Kp) even at lower amount of 5 µg (**Figure S4 A, C**). Ali et al., (2015) synthesized AgNPs synthesized from aqueous leaf extract of *Eucalyptus globules* exhibited antimicrobial action with the MIC against *E. coli* to be 36 µg/mL [42]. AgNPs synthesized from*Gracilaria dura* [43]*, Phormidiumformosum*[44]*, Anabaena doliolum*[45] *and Anabaena variabilis* [10] have also shown potent antibacterial properties. The antifungal activity of capped AgNPs exhibited activity against *Candida albicans* and *Candida glabrata* was compared with antifungal drug fluconazole. Highest zone of inhibition was observed against *Candida albicans* at 5 µg (**Figure S4C,D**). The antimicrobial mechanisms are widely accepted i.e. either through direct contact with microorganisms or by the release of silver ions [8]. It has been reported that AgNPs attaches to the cell

wall and consequently infiltrate cause membrane damage, that lead to leakage of cellular contents and result in microbial death [46]. It was also suggested that the penetration of the Ag nanoparticles occurs due to presence of charge on the surface of the nanoparticles and the organism surface which increases the permeability of the plasma membrane, effecting proper transport in and out of the cell, causing cell death [47]. Loo et al.,(2015) further ascribed AgNPs with binding to thiols in proteins and disruption of the bacterial respiratory chain leading to generation of reactive oxygen species (ROS) [48]. As the nanoparticles have high affinity with the phosphorous and sulphur like compounds, when it gets inside the cell, it affects the DNA by inhibiting its replication, proteins that inhibit the cellular metabolism of the cell causing the death of the microorganisms [49]. AgNPs also causes, transfer of electron in oxide state or in reduced state as it penetrates inside the cell by inactivating the enzyme due to release of hydrogen peroxide, causing cell death [50]. Thus, the action of Ag nanoparticles against pathogenic bacteria makes it an ideal candidate as antimicrobial agent.

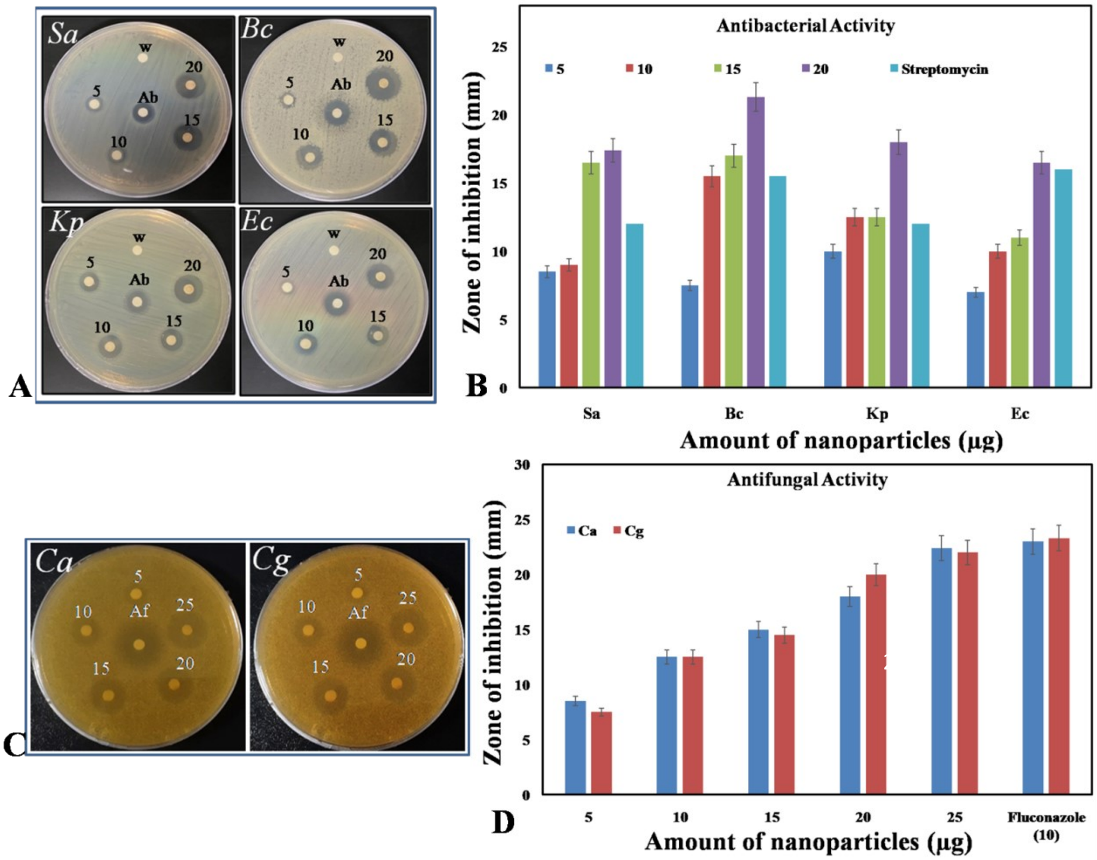

Figure S4. Experimental observations of the antibacterial property of *Plectonema*sp. NCCU-204-derived capped AgNPs;(**A,B**). Zone of inhibition with bar graph against bacterial pathogens:*Staphylococcus aureus* (Sa)*, Bacillus cereus* (Ba),*Klebsiella pneumoniae* (Kp) and *E. coli* (Ec). Antibiotic streptomycin was used as a positive control while water (w)

was used as a negative control for bacteria; (**C,D**) Zone of inhibition with bargraph of fungal pathogens *C. albicans* (Ca) and *C. glabrata* (Cg), antifungal (Af) fluconazolewas used as a positive control for fungus. Experimentswere performed in triplicates; mean ± SD are shown(p-value <0.05).

S5. In-vitro antioxidant activities of capped AgNPs

The phosphomolybdenum assay (total antioxidant capacity) of the reaction mixture was evaluated according to Prieto et al. (2005) [18]. Mixture containing 0.3 mL of aqueous extract (25 – 300 μL/mL) and AgNPs (25 – 175 μg/mL) was mixed with 1 mL of phosphomolybdate reagent (28 mM sodium phosphate, 4 mM ammonium molybdate and 0.6 M sulphuric acid). The test tubes containing the solution were incubated for 90 min at 95℃ in a water bath. The absorbance was measured at wavelength 695 nm against blank, 0.3 mL distilled water along with molybdate reagent using UV-visible spectrophotometer and ascorbic acid was used as reference standard. Experiment was performed in triplicates. Percentage of inhibition was calculated using the following formula:

4. $\% \text{ Inhibition/Scavenging} = \frac{(control\ absorbance - sample\ absorbance)}{control\ absorbance} \text{ X } 100$…………..Eq. (1)

DPPH (1, 1-diphenyl-2-picryl-hydrazil) free radical scavenging activity was performed by following the method of Gyamfi et al., (1999) [19]. Solution was prepared by dissolving 1mM DPPH in 80% ethanol which produces a solution of violet color. Further DPPH was added to ACE and AgNPs in 3:1 ratio by volume followed by incubation at 30℃ for 30 min in dark and absorbance was recorded at 517 nm against their respective blanks using double beam UV-visible spectrophotometer. Percentage of scavenging ability was calculated using equation 1 and IC_{50} value was obtained. Ascorbic acid was used as a reference standard. Experimental reactions were done in triplicates.

The ABTS (2,2'-azino-bis (3-ethylbenzothiazoline-6-sulfonic acid) free radicals scavenging activity was done using the modified protocol of Pellegrini et al. (1999) [20]. Adding 7mM ABTS solution to 2.45 mM potassium persulfate and reaction mixture was placed in dark at room temperature up to 16 h. Different concentrations of AgNPs (10 – 90 μg/mL) and extract (20 – 200 μg/mL) were added to ABTS separately then kept in dark for 10 min at 25 ℃. Absorbance was taken at 734 nm. ABTS alone was taken as blank while ascorbic acid was taken as positive control to compare the results. Eq. 1 was used to calculate scavenging activity.

Ferric reducing antioxidant power (FRAP) assayusing the method of Dinis et al., (1994) [21], fresh FRAP reagent was prepared by mixing 300 mM sodium acetate (pH 3.6), 10 mM TPTZ (Tripyridyltriazine) in 40 mM HCl and 20 mM $FeCl_3$ in 10:1:1 ratio at room temperature in the dark and

consumed immediately. 1.5 ml FRAP reagent was added to 200 µL of different concentration of AgNPs (50 – 450 µg/mL) and ACE (50 −450 µg/mL) following 4 min incubation and absorbance was taken at wavelength 593 nm. Ferrous sulfate was taken as a standard while ascorbic acid in this experiment was taken as a control. Experiment was done in triplicates.

The AgNP's nitric oxide radical scavenging activity was determined according to Ria et al., (2006) [22]. Mixture containing 500 µL of 5 mM sodium nitroprusside prepared in 0.1 M phosphate buffer (pH 7.4) was added to 1 mL of different concentration of aqueous cell extract (25−300 µg/mL) and AgNPs (10−120 µg/mL) and the reaction mixture was incubated for 30 min at 25 ℃. Further 500 µL of freshly prepared Griess reagent [1% sulphanilamide and 0.1% N-(1-Naphthyl) ethylenediamine] was added to the reaction mixture. Ascorbic acid was used as a positive control. The absorbance was measured at 546 nm using UV-visible spectrophotometer. Percentage of scavenging ability was calculated using Eq. 1.

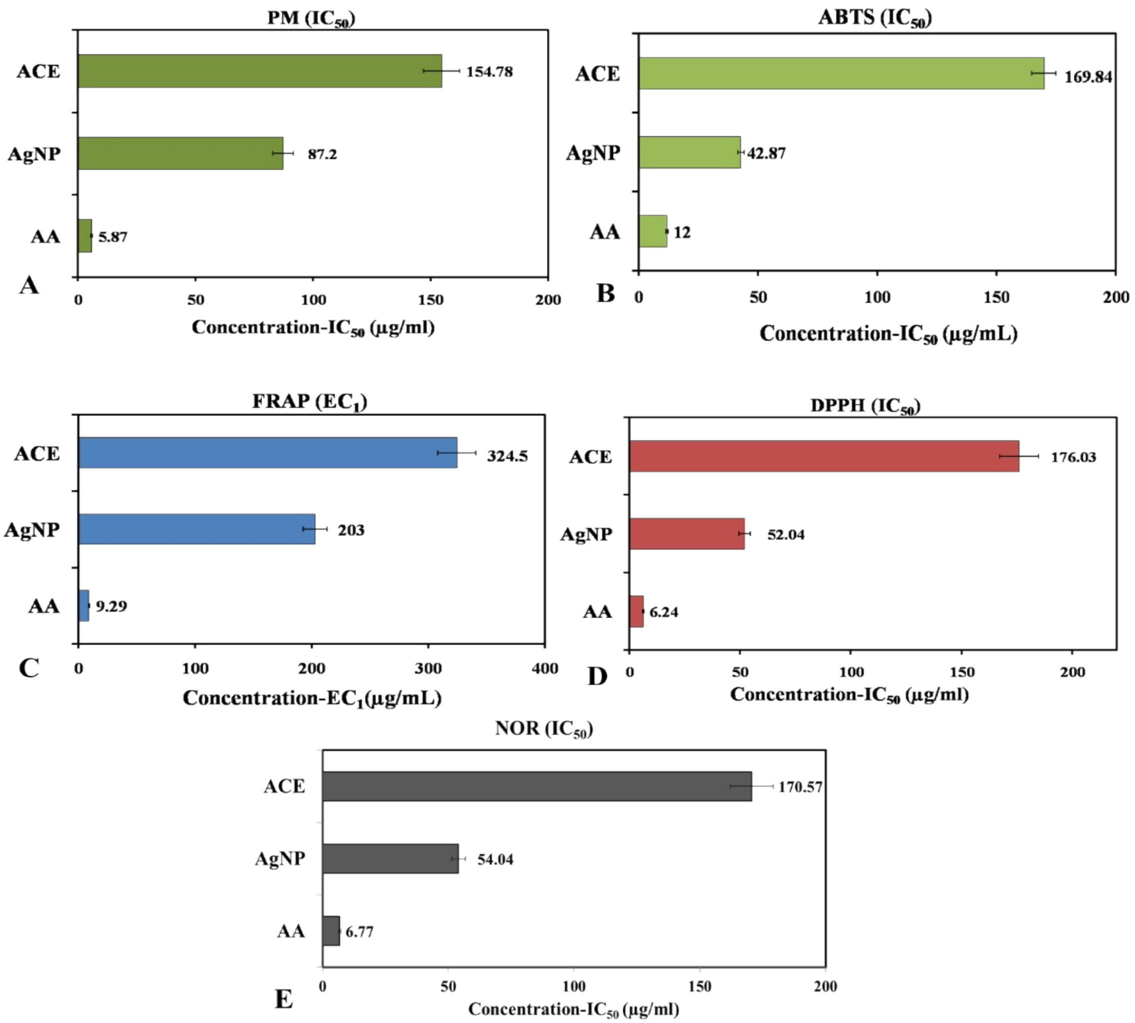

Figure S5. A. IC_{50} value for phosphomolybdenumassay B. IC_{50} value for ABTS assay C. EC_1 value for FRAP assay D. IC_{50} value for DPPH assay E. IC_{50} value for NOR assay.

S6. FTIR analysis of differently treated AgNPs

Table S2: FTIR analysis of capped and decapped (ethanol washed-SDS treated) nanoparticles

Wave number (cm^{-1})	Functional Group	Extract	Capped AgNPs (Water washed)	Capped AgNPs (Ethanol washed)	Capped AgNPs (Acetone washed)	DecappedAgNPs SDS treated	Decapped AgNPs Calcinated
**1045**	C-N stretching	Amine	Amine	Amine	Amine	Amine	-
**1211**	C-N stretching	Amine	Amine	Amine	Amine	_	-
**1381**	C-H bending	Alkanes (Dimethyl)	Alkanes (Dimethyl)	Alkanes (Dimethyl)	Alkanes (Dimethyl)	_	-
**1437**	O-H bending	Carboxylic acid	Carboxylic acid	Carboxylic acid	Carboxylic acid	_	-
**1508**	N-O stretching	Nitro compound	Nitro compound	Nitro compound	Nitro compound	_	-
**1623**	N-H bending	Amines	Amines	Amines	Amines	Amines	-
**2850**	N-H stretching	Amines	Amines	Amines	_	_	-
**2915**	N-H stretching	Amines	Amine	Amines	-	-	-
**3249**	O-H stretching	Alcohols	Alcohols	Alcohols	Alcohols	-	-

S7. LCMSMS ESI QTOF analysis for protein identification

S7.1. SDS-PAGE

Analysis was determined by using the protocol described by Laemmli, (1970) [15]. Different sample [aqueous cell extract (ACE), decappedAgNPs (SDS treated &calcinated) and capped AgNPs] were used to find out the role of protein in capped AgNPs synthesis present in the ACE and on the surface of nanoparticles. The samples were boiled with SDS solution (1%) for 15 min and then centrifuged at 12000 rpm for 13 min to collect the protein present in supernatant. The sample containing protein was mixed in ratio 1:1 with Laemmli sample buffer [0.5 M Tris−HCl (pH 6.8), 0.5% bromophenol blue, 10% glycerol, 5% β-mercaptoethanol, and 2% SDS] and incubated for 5 min at 100 ℃(water bath).The supernatant was used [after centrifugation (12000 rpm, 5 min and 4 ℃)] for electrophoresis on a mini protean gel system (Bio-Rad) at a constant voltage of 120 V at room temperature. After electrophoresis,

the gel was stained with 0.25% coomassie brilliant blue (CBB) R-250 in the 45% methanol−10% acetic acid solution for 3 h.

Further protein identification in capped AgNPs samples was analyzed by LCMSMS ESI QTOF (Chromous Biotech Pvt. Ltd., Bangalore). The samples were prepared by de-staining the coomassie stain gel (overnight in 50mM NH_4HCO_3 buffer in 10% Acetonitrile). Destained gel was subjected to overnight digestion with trypsin and the resulting peptides were extracted in 0.1% formic acid in water: 0.1% formic acid in acetonitrile (1:1 v/v). The extracted peptides were passed through ziptip for cleanup before MS analysis. About 10µL of sample was loaded on to the UHPLC instrument (model: ESI QTOF UPLC WATERS), and the protein separated on C18 column for 5min with the detection mass range of 100 to 2000 Da in Q1 scan mode. The mass data was analyzed on Mass Lynx software (Waters) and de-convoluted to single m/z masses. The masses obtained were taken for protein identification using in house MASCOT search engine with Swisprot database and Cyanobacterial taxonomy.

S7.2. LCMSMS ESI QTOF

S7.2.1. Equipment make/model: ESI QTOF UPLC WATERS

S7.1.2. LC Conditions: Column: 1.9um C8 2.1x50mm, Oven Temp: 40 ℃, Flow rate: 0.5 mL/min, Gradient Run time: 5 min, Buffer A: Water 0.1% Formic Acid (v/v), Buffer B: ACN 0.1% Formic Acid (v/v)

S7.1.3. MS Conditions: Source: ESI, Nebulizing Gas: 3L/min, Heating Gas Flow: 10L/min, Drying Gas Flow: 10L/min, Interface Temp: 300℃, DL Temp: 250℃, Heat Block: 400℃

S7.1.4. Results

S7.1.4.1. AgNPs – Upper protein band (~20KDa)

```
Database     : SwissProt 2018_05 (557491 sequences; 199978344 residues)
Taxonomy     : Cyanobacteria (13704 sequences)
Timestamp    : 27 Mar 2020 at 10:24:09 GMT
Top Score    : 88 for CHLP_SYNY3, Geranylgeranyl diphosphate reductase OS=Synechocystis sp. (strain PCC 6803 / Kazusa) OX=1111708 GN=chlP PE=3 SV=1
```

S7.1.4.1.1. Index

	Accession	Mass	Score	Description
1.	CHLP_SYNY3	45139	88	Geranylgeranyl diphosphate reductase OS=Synechocystis sp. (strain PCC 6803 / Kazusa) OX=1111708 GN=chlP PE=3 SV=1
2.	Y316_SYNS9	9621	30	UPF0367 protein Syncc9902_0316 OS=Synechococcus sp. (strain CC9902) OX=316279 GN=Syncc9902_0316 PE=3 SV=1
3.	SFSA_PROMT	30010	28	Sugar fermentation stimulation protein homolog OS=Prochlorococcus marinus (strain NATL2A) OX=59920 GN=sfsA PE=3 SV=1
4.	SSB_THEEB	13420	27	Single-stranded DNA-binding protein OS=Thermosynechococcus elongatus (strain BP-1) OX=197221 GN=ssb PE=3 SV=1
5.	TAL_PROM4	37641	25	Transaldolase OS=Prochlorococcus marinus (strain MIT 9211) OX=93059 GN=tal PE=3 SV=1
6.	SFSA_SYNJA	27350	25	Sugar fermentation stimulation protein homolog OS=Synechococcus sp. (strain JA-3-3Ab) OX=321327 GN=sfsA PE=3 SV=1
7.	PSTB_TRIEI	31150	24	Phosphate import ATP-binding protein PstB OS=Trichodesmium erythraeum (strain IMS101) OX=203124 GN=pstB PE=3 SV=1
8.	RL20_ANAVT	13507	23	50S ribosomal protein L20 OS=Anabaena variabilis (strain ATCC 29413 / PCC 7937) OX=240292 GN=rplT PE=3 SV=1
9.	AROC_PROMA	39747	23	Chorismate synthase OS=Prochlorococcus marinus (strain SARG / CCMP1375 / SS120) OX=167539 GN=aroC PE=3 SV=2
10.	AROC_SYNE7	39411	23	Chorismate synthase OS=Synechococcus elongatus (strain PCC 7942) OX=1140 GN=aroC PE=3 SV=1
11.	AROC_SYNP6	39469	23	Chorismate synthase OS=Synechococcus sp. (strain ATCC 27144 / PCC 6301 / SAUG 1402/1) OX=269084 GN=aroC PE=3 SV=1
12.	SYK_GLOVI	56094	22	Lysine--tRNA ligase OS=Gloeobacter violaceus (strain PCC 7421) OX=251221 GN=lysS PE=3 SV=1
13.	RF1_SYNJA	40930	22	Peptide chain release factor 1 OS=Synechococcus sp. (strain JA-3-3Ab) OX=321327 GN=prfA PE=3 SV=1
14.	MOAA_SYNPX	38031	22	GTP 3',8-cyclase OS=Synechococcus sp. (strain WH8102) OX=84588 GN=moaA PE=3 SV=1
15.	BDLP_NOSP7	78699	21	Bacterial dynamin-like protein OS=Nostoc punctiforme (strain ATCC 29133 / PCC 73102) OX=63737 GN=Npun_R6513 PE=1 SV=1
16.	MOAA_CYAP4	37650	21	GTP 3',8-cyclase OS=Cyanothece sp. (strain PCC 7425 / ATCC 29141) OX=395961 GN=moaA PE=3 SV=1
17.	RPOZ_THEEB	8906	21	DNA-directed RNA polymerase subunit omega OS=Thermosynechococcus elongatus (strain BP-1) OX=197221 GN=rpoZ PE=3 SV=1
18.	AROC_PROM1	39148	21	Chorismate synthase OS=Prochlorococcus marinus (strain NATL1A) OX=167555 GN=aroC PE=3 SV=1
19.	PHNC3_NOSS1	28888	20	Phosphonates import ATP-binding protein PhnC 3 OS=Nostoc sp. (strain PCC 7120 / SAG 25.82 / UTEX 2576) OX=103690 GN=phnC3 P
20.	RL20_NOSP7	13497	20	50S ribosomal protein L20 OS=Nostoc punctiforme (strain ATCC 29133 / PCC 73102) OX=63737 GN=rplT PE=3 SV=1

S7.1.4.1.2.Peptide Mass Spectra: AgNPs – Upper protein band (~20KDa)

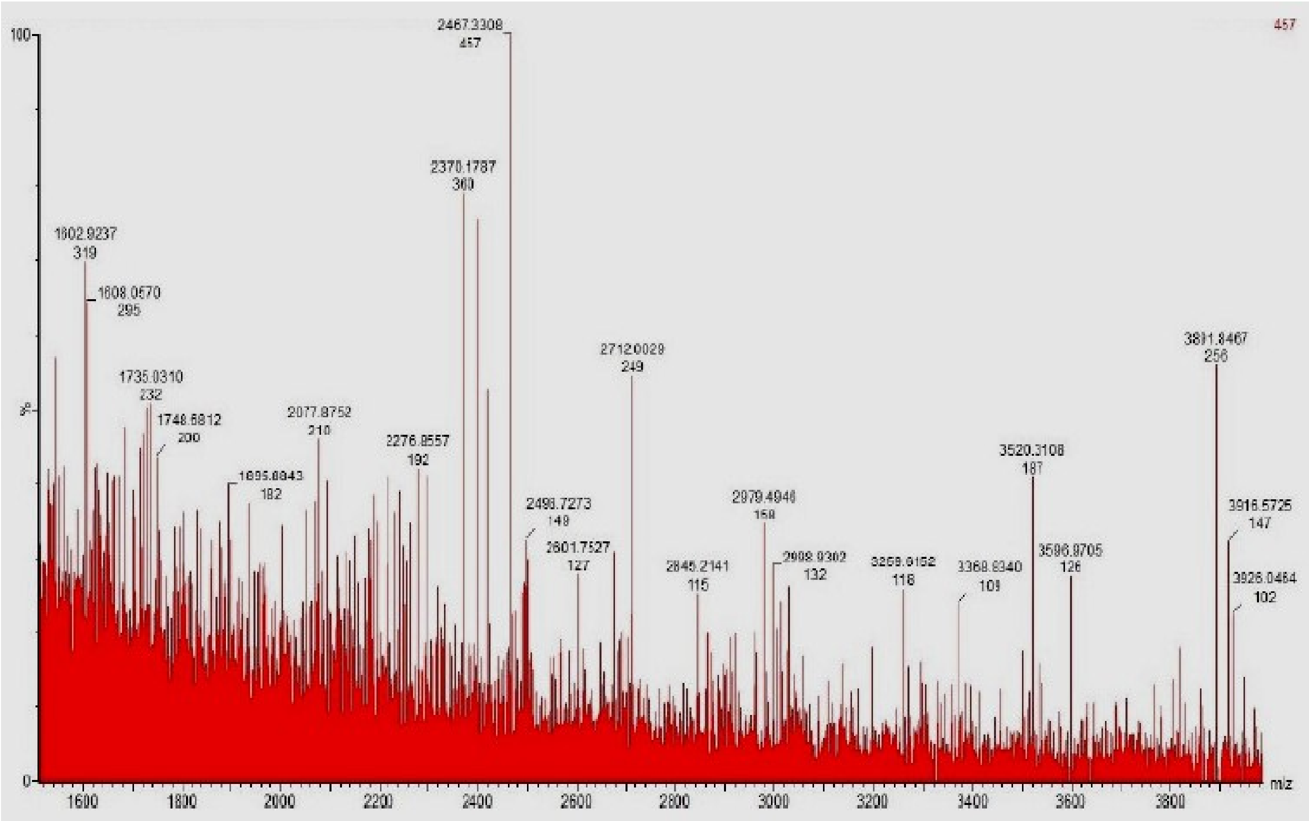

S7.1.4.2. AgNPs: Lower protein band (~12 KDa)

Database	: SwissProt 2018_05 (557491 sequences; 199978344 residues)
Taxonomy	: Cyanobacteria (13704 sequences)
Timestamp	: 27 Mar 2020 at 12:02:57 GMT
Top Score	: 130 for LEU1_PROMP, 2-isopropylmalate synthase OS=Prochlorococcus marinus subsp. pastoris (strain CCMP1986 / NIES-2087 / MED4) OX=59919

S7.1.4.2.1. Index

	Accession	Mass	Score	Description
1.	LEU1_PROMP	59610	130	2-isopropylmalate synthase OS=Prochlorococcus marinus subsp. pastoris (strain CCMP1986 / NIES-2087 / MED4) OX=59919 GN=leuA
2.	LEU1_PROM5	59749	68	2-isopropylmalate synthase OS=Prochlorococcus marinus (strain MIT 9515) OX=167542 GN=leuA PE=3 SV=1
3.	LEU1_PROM0	59482	68	2-isopropylmalate synthase OS=Prochlorococcus marinus (strain MIT 9301) OX=167546 GN=leuA PE=3 SV=1
4.	LEU1_PROM9	59616	64	2-isopropylmalate synthase OS=Prochlorococcus marinus (strain MIT 9312) OX=74546 GN=leuA PE=3 SV=1
5.	LEU1_PROMS	59445	62	2-isopropylmalate synthase OS=Prochlorococcus marinus (strain AS9601) OX=146891 GN=leuA PE=3 SV=1
6.	LEU1_PROM2	59495	53	2-isopropylmalate synthase OS=Prochlorococcus marinus (strain MIT 9215) OX=93060 GN=leuA PE=3 SV=1
7.	ILVC_SYNPW	35950	28	Ketol-acid reductoisomerase (NADP(+)) OS=Synechococcus sp. (strain WH7803) OX=32051 GN=ilvC PE=3 SV=1
8.	RF1_ANAVT	41322	26	Peptide chain release factor 1 OS=Anabaena variabilis (strain ATCC 29413 / PCC 7937) OX=240292 GN=prfA PE=3 SV=1
9.	MINC_PROMM	24841	26	Probable septum site-determining protein MinC OS=Prochlorococcus marinus (strain MIT 9313) OX=74547 GN=minC PE=3 SV=1
10.	RF1_NOSS1	41324	24	Peptide chain release factor 1 OS=Nostoc sp. (strain PCC 7120 / SAG 25.82 / UTEX 2576) OX=103690 GN=prfA PE=3 SV=1
11.	RS13_SYNY3	14563	24	30S ribosomal protein S13 OS=Synechocystis sp. (strain PCC 6803 / Kazusa) OX=1111708 GN=rpsM PE=3 SV=1
12.	PSBX_ANAVT	4329	24	Photosystem II reaction center X protein OS=Anabaena variabilis (strain ATCC 29413 / PCC 7937) OX=240292 GN=psbX PE=3 SV=1
13.	PSBX_NOSS1	4329	24	Photosystem II reaction center X protein OS=Nostoc sp. (strain PCC 7120 / SAG 25.82 / UTEX 2576) OX=103690 GN=psbX PE=3 SV=1
14.	ILVC_SYNPX	35975	24	Ketol-acid reductoisomerase (NADP(+)) OS=Synechococcus sp. (strain WH8102) OX=84588 GN=ilvC PE=3 SV=1
15.	ILVC_PROMA	36036	24	Ketol-acid reductoisomerase (NADP(+)) OS=Prochlorococcus marinus (strain SARG / CCMP1375 / SS120) OX=167539 GN=ilvC PE=3 SV=
16.	RS13_CYAP8	14536	23	30S ribosomal protein S13 OS=Cyanothece sp. (strain PCC 8801) OX=41431 GN=rpsM PE=3 SV=1
17.	RL6_SYNS9	19041	23	50S ribosomal protein L6 OS=Synechococcus sp. (strain CC9902) OX=316279 GN=rplF PE=3 SV=1
18.	RS13_MICAN	14412	23	30S ribosomal protein S13 OS=Microcystis aeruginosa (strain NIES-843) OX=449447 GN=rpsM PE=3 SV=1
19.	YCF4_THEEB	20929	23	Photosystem I assembly protein Ycf4 OS=Thermosynechococcus elongatus (strain BP-1) OX=197221 GN=ycf4 PE=3 SV=1
20.	COAD_CYAP4	18900	22	Phosphopantetheine adenylyltransferase OS=Cyanothece sp. (strain PCC 7425 / ATCC 29141) OX=395961 GN=coaD PE=3 SV=1

S7.1.4.2.2. Peptide Mass Spectra: AgNPs- Lower protein band (~12 KDa)

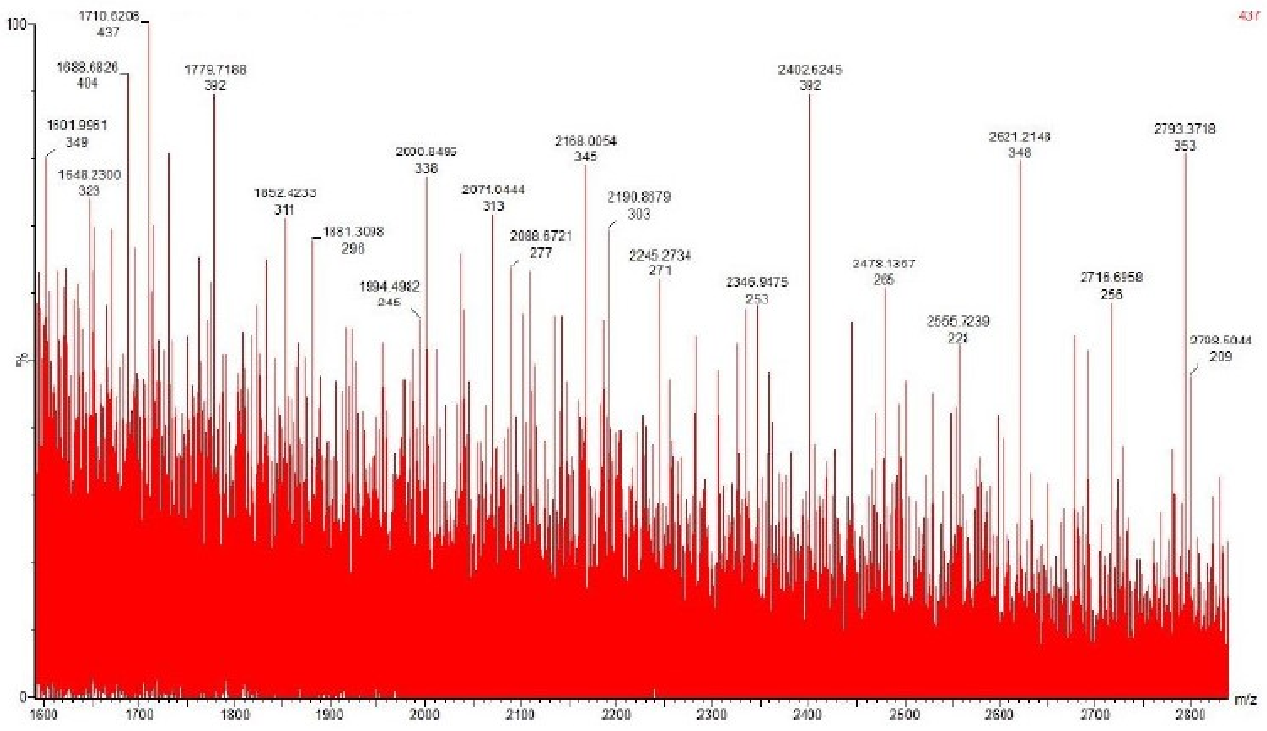

S8. Antibacterial activity of capped and decapped(ethanol washed-SDS treated) AgNPs

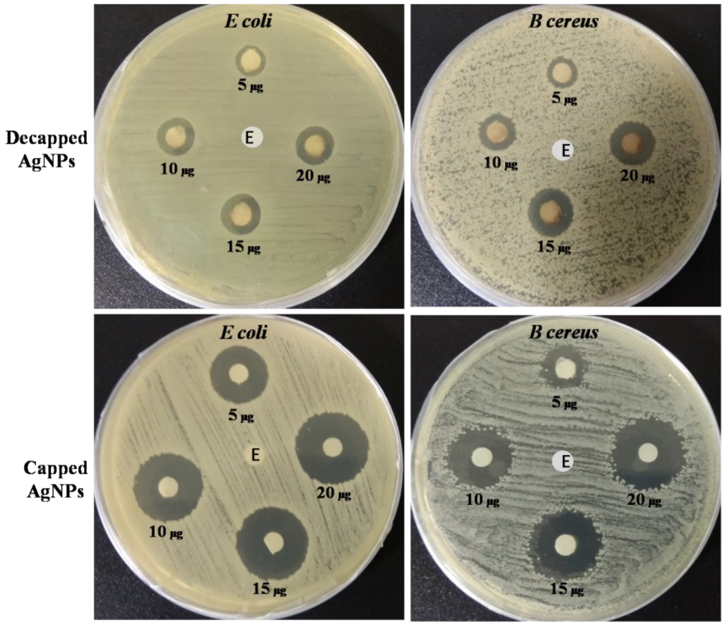

Figure S6. Antibacterial activity of capped and decapped(ethanol washed-SDS treated) AgNPs

S9. Total protein content

Modified method of Lowry et al. 1951 was used to estimate of total protein. In a test tube 1 mL NaOH (1N) was added to 1 mL test samples and placed in boiling water bath for 10 min and cooled under tap water. For standard curve, a blank containing 1 mL ddH_2O and 1 mL each concentrations (25, 50 100, 150, 200, 250, 300 mg/mL) of BSA solution were taken. 5 mL of Reagent A (prepared by adding 1 mL freshly prepared 1% Na-K Tartarate solution containing 0.5% $CuSO_4$ into 50 mL 2% Na_2CO_3 solution) was added to standard/sample/blank and incubated at room temperatures for 10 min. Reagent B (Folin's reagent) 0.5 mL was added to the reaction mixture and further incubated at room temperature for 15 min. The absorbance was observed at λ=650 nm against blank. Protein content was evaluated from the standard of BSA solution with known concentration. Experiment was carried out in triplicates.

S10. Biocompatibility assay of capped and decapped AgNPs

Peripheral blood mononuclear cell (PBMC) was taken from fresh human blood (20-25 ml) received from Ansari Health Centre, Jamia Millia Islamia, New Delhi, India. The blood sample was diluted with the same phosphate buffered saline (PBS) volume and carefully layered on Ficoll-Histopaque (Sigma Aldrich, USA). The mixture was centrifuged under at 400g for 30 min at 20–22 °C. The unperturbed pellet (lymphocyte layer) was carefully transferred out. The lymphocyte was washed and pelleted down with three volumes of PBS for twice and resuspended RPMI-1640 media (Gibco, USA) with 10% antibiotic and antimycotic solution (Gibco, USA) and v/v fetal calf serum (FCS) (Gibco, USA). Cell counting was performed to determine the PBMC cell number with equal volume of trypan blue.

RPMI-1640 culture medium with 10% heat-inactivated fetal calf serum and antibiotic antimycotic solution was used to culture A549 (non-small cell lung cancer cell lines). A fixed number of cells with density of $7*10^3$ cells/well were seeded in a 96-well microtiter plate and treated with capped and de-capped AgNPs for 48 h. Afterward, 20 µL of 5mg/mL of MTT [3-(4, 5-dimethylthiazolyl-2)-2, 5-diphenyltetrazolium bromide] dye was added to each well. The MTT dye was reduced by mitochondrial succinate dehydrogenase of live cells to an insoluble, coloured formazan product [28]. The formazan was then solubilised in 100 µl of DMSO. The resulting color was read at 570 nm on ELISA plate reader (iMark, BIO-RAD). The percentage of viable cell was calculated as:

$$\% \text{ of viable cells} = \frac{A_T - A_B}{A_C - A_B} \times 100 \qquad \text{……………. Eq 3}$$

Where A_C = absorbance of the control (mean value): A_T = absorbance of the treated cells (mean value), A_B = absorbance of the blank (mean value). IC_{50} values of Ag NPs were determined for each cell line.

Cells were stained with fluorescent 4',6-diamidino-2-phenylindole (DAPI) to detect nuclear condensation and blebbing. After 48 hr of treatment, the cells in a 12 well plate were washed with PBS thrice. Cells were then fixed with 4% paraformaldehyde for 8 min and then washed with PBS thrice. After that, the cells were permeabilized by treating with 0.1% Triton X-100 for 2 min. Again washed 3 times with PBS and treated with fluorescent dye DAPI 1µg/ml. After 5 min PBS was added to the well to keep the cells hydrated while imaging under fluorescent microscope ZOE Fluorescent Cell Imager, Bio-Rad.
